# From Oxidative Stress Damage to Pathways, Networks, and Autophagy via MicroRNAs

**DOI:** 10.1155/2018/4968321

**Published:** 2018-04-12

**Authors:** Nikolai Engedal, Eva Žerovnik, Alexander Rudov, Francesco Galli, Fabiola Olivieri, Antonio Domenico Procopio, Maria Rita Rippo, Vladia Monsurrò, Michele Betti, Maria Cristina Albertini

**Affiliations:** ^1^Nordic EMBL Partnership for Molecular Medicine, Centre for Molecular Medicine Norway (NCMM), University of Oslo, P.O. Box 1137, Blindern, 0318 Oslo, Norway; ^2^Department of Biochemistry and Molecular and Structural Biology, Jožef Stefan Institute and Center of Excellence for Integrated Approaches in Chemistry and Biology of Proteins (CipKeBip), Ljubljana, Slovenia; ^3^Department of Biomolecular Sciences, University of Urbino “Carlo Bo”, Urbino, Italy; ^4^Laboratory of Clinical Biochemistry and Nutrition, Department of Pharmaceutical Sciences, University of Perugia, Perugia, Italy; ^5^Department of Molecular and Clinical Sciences, Marche Polytechnic University, Ancona, Italy; ^6^Center of Clinical Pathology and Innovative Therapy, Italian National Research Center on Aging INRCA-IRCCS, Ancona, Italy; ^7^Department of Medicine, University of Verona, Verona, Italy

## Abstract

Oxidative stress can alter the expression level of many microRNAs (miRNAs), but how these changes are integrated and related to oxidative stress responses is poorly understood. In this article, we addressed this question by using in silico tools. We reviewed the literature for miRNAs whose expression is altered upon oxidative stress damage and used them in combination with various databases and software to predict common gene targets of oxidative stress-modulated miRNAs and affected pathways. Furthermore, we identified miRNAs that simultaneously target the predicted oxidative stress-modulated miRNA gene targets. This generated a list of novel candidate miRNAs potentially involved in oxidative stress responses. By literature search and grouping of pathways and cellular responses, we could classify these candidate miRNAs and their targets into a larger scheme related to oxidative stress responses. To further exemplify the potential of our approach in free radical research, we used our explorative tools in combination with ingenuity pathway analysis to successfully identify new candidate miRNAs involved in the ubiquitination process, a master regulator of cellular responses to oxidative stress and proteostasis. Lastly, we demonstrate that our approach may also be useful to identify novel candidate connections between oxidative stress-related miRNAs and autophagy. In summary, our results indicate novel and important aspects with regard to the integrated biological roles of oxidative stress-modulated miRNAs and demonstrate how this type of in silico approach can be useful as a starting point to generate hypotheses and guide further research on the interrelation between miRNA-based gene regulation, oxidative stress signaling pathways, and autophagy.

## 1. Introduction

The flux and redox chemistry of reactive oxygen species (ROS) influence key physiological responses of tissues through the capacity of being able to regulate virtually all signal transduction pathways and gene transcription factors of the cellular systems. As a consequence, disturbances of the regulatory role of ROS, often described with the generic term “oxidative stress,” can lead to the development of major cellular failures that have been described as a recurring trait in the pathobiology of many, if not all, types of diseases.

The increasing interest in control mechanisms of the gene-environment interaction has stimulated a series of studies in this field, pointing to microRNA (miRNA) molecules as emerging molecular mediators of oxidative stress and ROS chemistry. Moreover, accumulating evidence points to a central role of the lysosomal degradative pathway autophagy in oxidative stress responses and in oxidative stress-related pathobiology.

This article focuses on the relations between oxidative stress, microRNAs, and autophagy. We use existing knowledge combined with in silico analyses to introduce concepts that can be useful for studying the connections between miRNA-based gene regulation, oxidative stress-induced pathways, and autophagy.

miRNAs are small noncoding RNAs, which, after a process of maturation, have a typical length of 18–25 bp. miRNAs have a unique role in posttranscriptional gene regulation. Depending on various grades of complementarities, miRNAs can cause a block in mRNA translation or even mRNA degradation. Since their discovery, miRNAs have been known to regulate the expression of a very large number of proteins, and it is supposed that they could regulate up to 30% (or even more) of the human genome [1]. Different studies show the importance of miRNAs in the regulation of processes like cell growth, differentiation, apoptosis, and carcinogenesis. Furthermore, miRNAs can be expected to play an important role in the diagnosis and prognosis of a large number of human diseases, since the quali-quantitative miRNA composition of every tissue is different depending on the state of human health [2].

In the modern way of defining oxidative stress, this adverse condition occurs when a cell or tissue is unable of controlling redox-dependent reactions and signal transduction processes by modified flux or reactivity of ROS. Depending on the intensity of this redox challenge, biomolecule damage may also occur, with accumulation of byproducts and increased need for detoxification and turnover of cellular components. The generic term “ROS” is used to comprehensively describe a series of molecules that derive from the tetravalent reduction of molecular oxygen and NO-derived metabolites, plus a series of second-generation products of their reactivity with biomolecules (lipids, proteins, and nucleic acids). Second-generation products include amongst others organic free radicals, peroxides, and reactive carbonyls, which are reported to play important roles in aging and disease development. The most relevant ROS forms in cellular systems are mainly represented by hydrogen peroxide and superoxide anion, which play important biochemical roles spanning from cell cycle regulation to the defense against pathogens during phagocytosis. Redox homeostasis, and thus the physiological control of redox-sensitive signal transduction pathways, is ensured by the activity of a battery of cellular detoxification and antioxidant enzymes.

Since miRNAs are important modulators of protein expression, the functional relationship between oxidative stress and the miRNA-dependent regulation of ROS-generating and redox-regulating enzymes, and their associated targets and pathways, is of great interest. However, the interaction between miRNAs and their molecular targets is often complex and difficult to interpret, thus introducing a major technical complication in explorative and prediction studies as well as in model interpretation. Indeed, every single gene target can be regulated by many miRNAs and every single miRNA may regulate the expression of many different target proteins [2].

Several types of dedicated software for consultation of miRNA databases are available on the web, and this helps to get a comprehensive overview of possible interactions. In recent years, different algorithms have been developed to predict the role of miRNAs expressed in different organisms, which besides humans include other vertebrates, *Drosophila melanogaster*, and plants. The database DIANA LAB [3–6] includes various algorithms that predict the association between a miRNA and its targets, analysis of expression data, and pathway attribution. Other web resources are MicroInspector [7], miRanda-mirSVR [8, 9], NBmiRTar [10], PicTar [11], Segal Lab of Computational Biology [12], RNA22 microRNA target detection [13], and TargetScan [13–15]. Another very useful web resource is miRecords [16], a collection of experimentally validated miRNA-target interactions. These resources helped us to develop a program called SID1.0 (String IDentifier) able to associate the targets and pathways of different miRNAs, and even in the opposite way, to associate them to different miRNAs [17].

In the present study, we firstly identified a small number of miRNAs already observed in the literature to be up- or downregulated after exposure of *in vitro* cultured human cells to oxidative stress. Further, using TargetScan [13–15] and DIANA LAB [6], we searched for the targets and pathways of those miRNAs implicated in oxidative stress, before employing SID 1.0 [17] for final determination of the common targets and pathways. We used miRecords [16] to identify the data already validated experimentally. Next, we searched for new miRNAs likely associated to the targets and pathways found to be involved in the oxidative stress response. Moreover, we analyzed the targets obtained from SID1.0 through the use of Ingenuity Pathways Analysis (IPA; Ingenuity® Systems, http://www.ingenuity.com) to show the networks and biological functions likely modulated by the identified targets. One of the most dominant pathways turned out to be protein ubiquitination. Lastly, we performed a literature search for links between our identified target genes, as well as our identified candidate novel oxidative stress-related miRNAs, and autophagy.

## 2. Materials and Methods

### 2.1. Data Acquisition and SID1.0 Prediction Analysis

From the TargetScan database, we obtained the predicted target genes of the miRNAs of interest. The targets of a miRNA are indicated with a specific gene ID system (RefSeq ID). For each miRNA, a dataset (i.e., a group list of RefSeq IDs) of the predicted targeted genes was created. Since a visual inspection of the IDs would be impractical due to their large number (up to thousands of IDs), they have been automatically indexed using a simple program written in Fortran (SID1.0; String IDentifier, see http://www.fis.uniurb.it/spada/SID_minipage.html and [17]) that looks for RefSeq IDs shared by the predicted target genes of the different datasets. SID1.0 is in fact based on an algorithm of sequential exhaustive searches that has been implemented in Fortran 90 using very elementary methods. SID1.0 performs an exhaustive search within each individual one-column ASCII input file and reports the result (i.e., the number of common targets) on an ASCII output file in the form of a table that summarizes the common IDs. Thus, the main advantage of SID1.0, which works as a filter on the information provided by the web pages hosting the miRNA databases, is that it is completely independent from the algorithms on which the databases rely. In this way, our procedure builds upon the prediction algorithms used in the databases, whose outputs are scrutinized by SID1.0. SID1.0 has been developed and tested in a Mac OS X environment and is currently compiled using the Intel Fortran 90 compiler.

Each gene in the group list has the related information in NCBI's Entrez Nucleotide database. It is possible to perform a reverse search by obtaining the miRNAs predicted to target a gene from TargetScan. For each gene, a dataset of the miRNAs predicted to target the gene was created. The names of the miRNAs were indexed using SID1.0, which looks for miRNA names shared by the predicted targeting miRNAs of the different datasets. Furthermore, for a defined miRNA name, target genes can be automatically retrieved from the DIANA-microT 3.0 database. A list of gene names or a list of RefSeq IDs is provided, and the program translates them into Ensembl IDs. The list of genes is compared to the Kyoto Encyclopedia of Genes and Genomes (KEGG) Pathways Database, and IDs are indexed using SID1.0, which looks for KEGG pathway IDs shared by the predicted target genes of the different datasets.

In this way, we were able to obtain the common target genes of specific miRNAs, the common targeting miRNAs of specific genes, and the common pathways of specific miRNAs.

### 2.2. IPA (Ingenuity Pathways Analysis)

Data were analyzed by the use of Ingenuity Pathways Analysis (Ingenuity Systems, http://www.ingenuity.com).

For a graphical representation of the molecular relationships between selected target molecules involved in oxidative stress, molecules are represented as nodes, and the biological relationship between two nodes is represented as an edge (line). All edges are supported by at least 1 reference from the literature, from a textbook, or from canonical information stored in the Ingenuity Pathways Knowledge Base.

The functional analysis identified the biological functions that were most significant to the molecules in the network. The network molecules associated with biological functions in Ingenuity's Knowledge Base were considered for the analysis. Right-tailed Fisher's exact test was used in assigning each biological function to a particular network.

## 3. Results and Discussion

### 3.1. Oxidative Stress-Modulated miRNAs

From the literature, we found the following 13 miRNAs to be modulated by oxidative stress in human cultured cells: let-7f, miR-9, miR-16, miR-21, miR-22, miR-29b, miR-99a, miR-125b, miR-128, miR-143, miR-144, miR-155, and miR-200c [18–23]. For the research on oxidative stress-induced alterations of miRNA expression, many studies used H_2_O_2_. For example, Simone et al. [18] have shown that H_2_O_2_-treated AG01522 primary human fibroblasts alter their expression of let-7f, miR-16, miR-21, miR-22, miR-99a, miR-143, and miR-155. Magenta et al. [19] used human umbilical vein endothelial cells (HUVEC) treated with H_2_O_2_, and microRNA profiling showed an increased miR-200c expression. Sangokoya et al. [20] used different H_2_O_2_ concentrations to treat K562 erythroleukemia cells, which responded with increased expression of miR-144. Worth of note, in this study miR-144 was identified to, in both K562 cells and primary erythroid progenitor cells, directly regulate the activity of nuclear factor-erythroid 2-related factor 2 (Nrf2 or NFE2L2), a transcription factor and master regulator of detoxification and antioxidant responses [20].

Other studies used different agents to induce ROS generation: for example, Kutty et al. [21] used *N*-(4-hydroxyphenyl)retinamide (4HPR), a retinoic acid derivative and ROS-generating agent, and showed that 4HPR increases the expression of miR-9 in human retinal pigment epithelial (ARPE-19) cells. Luna et al. [22] induced chronic oxidative stress in HTM cells (human trabecular meshwork cells) by incubation at 40% oxygen compared to 5% oxygen control-treated cells. In response to this, miR-29b expression was decreased, and since miR-29b regulates extracellular matrix (ECM) expression it could indicate that miR-29b downregulation was responsible for increased expression of several ECM genes after oxidative stress. The combination of iron and aluminum sulfate is known to produce ROS in cultures of human brain HN cells. Lukiw and Pogue [23] isolated microRNAs from HN cells exposed to magnesium sulfate (control), aluminum sulfate, or aluminum plus iron sulfate. microRNA arrays showed that miR-9, miR-125b, and miR-128 were upregulated by metal sulfate-generated ROS.

### 3.2. Common Targets to Oxidative Stress-Modulated miRNAs

Using our predicting tool SID1.0 [17], we identified common target genes of the 13 oxidative stress-modulated miRNAs described in the previous paragraph (let-7f, miR-9, miR-16, miR-21, miR-22, miR-29b, miR-99a, miR-125b, miR-128, miR-143, miR-144, miR-155, and miR-200c) (Table 1 and Supplementary Table 1).

We did not find any target gene common to all oxidative stress-modulated miRNAs, but we identified 13 target genes that were common to 5, 6, or 7 of them (Table 1 and Supplementary Table 1). Using miRecords [16], we found that out of these 13 targets, 3 genes (CDC14B, NFIB, and PPARA; highlighted in italics in Table 1 and bold in Supplementary Table 1) were targets that have been experimentally validated for interaction with one of the 13 oxidative stress-modulated miRNAs. Moreover, and intriguingly, the products of these 3 genes have been described to be involved in oxidative stress damage responses. CDC14B (CDC14 cell division cycle 14 homolog B) is a member of the dual-specificity protein tyrosine phosphatase family. Its protein expression has been validated to be modulated by miR-16 and miR-15b. CDC14B is involved in cell cycle control, inducing the exit of cell mitosis and initiation of DNA replication. In response to genotoxic stress, it can translocate to the nucleoplasma to activate the ubiquitin ligase APC/C (Cdh1) and, via a number of events, promote a G2 DNA damage response checkpoint [24–26]. NFIB (nuclear factor I/B) induces in association with MYB the expression of various proteins implicated in apoptosis, cell growth, cell cycle control, and cell adhesion. NFIB is a negative regulator of miR-21, as it binds the miR-21 promoter. Interestingly, it is on the other hand the NFIB mRNA that has been validated to be regulated by miR-21, thus constituting a form of double-negative feedback system [27, 28]. PPARA (peroxisome proliferator-activated receptor alpha) is a transcription factor of the steroid hormone receptor family. It regulates the expression of target genes implicated in cell proliferation, cell differentiation, and immune and inflammation responses. It has been shown that ROS induce the expression of PPARA [29]. Further, it has been validated that the expression of PPARA is regulated by miR-22 [16].

Interestingly, 5 of the other targets found in our analysis (SH3PXD2A, CBL, ClCN5, USP31, and LIFR) have been indirectly implicated in oxidative stress responses. SH3PXD2A (SH3 and PX domains 2A, also called Tks5) has been described to link NOX (NADPH oxidases) to ROS formation [30]. CBL (Cas-Br-M (murine) ecotropic retroviral transforming sequence), a ubiquitously expressed cytoplasmic adaptor protein, is simultaneously involved in the rapid degradation of TRAIL receptors and Akt phosphorylation during TRAIL treatment. Akt catalytic activation is known to increase during metabolic oxidative stress [31, 32]. Lack of proximal tubule ClCN5 is associated with increased cell proliferation and oxidative stress in mice and men [33]. LIFR (leukemia inhibitory factor receptor alpha) and its ligands play an essential role in endogenous neuroprotective mechanisms triggered by preconditioning-induced stress [34, 35]. Ubiquitin-specific peptidase 31, USP31, has a role in the regulation of NF-*κ*B activation (implicated in stress response) by members of the TNF receptor superfamily [36].

The remaining 5 targets identified by our prediction analysis (ZNF618, TNRC6B, CPEB3, KCNA1, and tcag7.1228; see Table 1 for annotations) are novel candidate gene products associated with oxidative stress responses yet to be experimentally explored.

For a full list of gene targets found to be common to ≥2 of the 13 oxidative stress-modulated miRNAs (i.e., all possible combinations), see Supplementary Table 2.

### 3.3. Common Pathways of Oxidative Stress-Modulated MicroRNAs

Using the DIANA mirPath database (DIANA LAB), we were able to identify the common pathways of the oxidative stress-modulated miRNAs analyzed above. The analysis revealed 25 pathways that were common to all 13 oxidative stress-modulated miRNAs (Table 2 and Supplementary Table 3).

**Figure 1:**
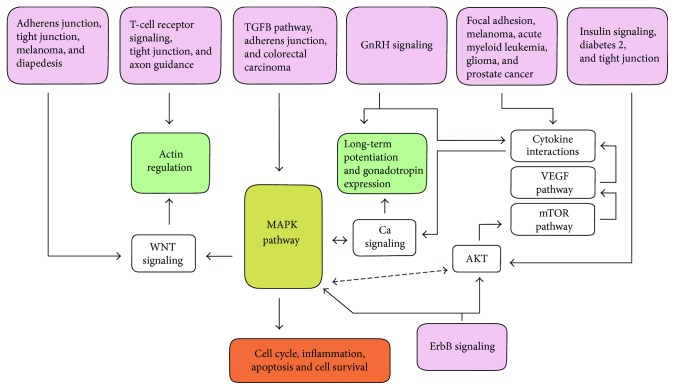
Simplified flow diagram indicating the interrelation between pathways predicted to be commonly modified by the 13 oxidative stress-related miRNAs considered in this study.

Confirming the validity of our analyses, most of these pathways are known to be involved in oxidative stress responses. Amongst the pathways predicted, many important cellular functions can be mentioned. For example, the MAPK signaling pathway entails a group of important signal transduction pathways involved in various cellular functions, including cell proliferation, differentiation, and migration. In fact, it is related to almost all of the other predicted pathways (Figure 1).

The calcium signaling pathway includes a group of events leading to increased cytosolic Ca^2+^ concentrations from extra- and intracellular (ER) sources. It is also one of the basic cellular signaling pathways implied in a wide range of cellular functions. Two pathways (cytokine-cytokine receptor interaction and TGF-beta signaling pathway) are related to cytokines, which are important intercellular messengers and regulators involved in inflammatory defenses, cell growth and differentiation, apoptosis, angiogenesis, and homeostasis. Two pathways are related to the immune system (T cell receptor signaling pathway, leukocyte transendothelial migration) and are responsible for the activation of T-lymphocytes and for the transendothelial migration of leukocytes from the blood to the tissues. Five pathways are related to the cytoskeleton, extracellular matrix, and cell-cell and cell-matrix adhesion. They include adherens junctions (cell-cell adhesion), epithelial tight junctions, focal adhesions (cell-matrix adhesion), cell adhesion molecules (selectins, cadherins, integrins, and immunoglobulins) involved in cellular adhesion, costimulation, and antigen recognition, and one actin cytoskeleton regulation pathway. Two pathways include the insulin signaling pathway, leading to glycogen synthesis and increased glucose uptake, and the related type II diabetes mellitus, leading to insulin resistance through inhibition of IRS1 functions. The GnRH signaling pathway is leading to gonadotropin-releasing hormone secretion and regulation of the production and release of the gonadotropins by the pituitary. The VEGF signaling pathway is highly important in angiogenesis and is regulating a variety of very different endothelial/epithelial processes, such as proliferation and migration of endothelial cells, promotion of epithelial survival, and vascular permeability. The Wnt signaling pathway is responsible for cell fate decisions, progenitor cell proliferation, and control of asymmetric cell division in different tissues. Two pathways are related to neuronal network development. Axon guidance is important for the development of the neuronal network, and long-term potentiation is the molecular basis for learning and memory. Six pathways are related to cancers and leukemia and more specifically to acute myeloid leukemia, prostate cancer, colorectal cancer, glioma (brain tumor), and skin cancer (melanoma and basal cell carcinoma).

Interestingly, three components of the above-mentioned pathways have been validated to be modulated by the oxidative stress-modulated miRNAs of interest: TGF-beta receptor type II (TGFBR2), implied in MAPK- and TGF-beta signaling pathways, has been validated to be regulated by miR-21 (and miR-26a). Interestingly, TGFBR2 has also been related to the production of ROS [37, 38]. CDH1 is implied in melanoma, adherens junctions, and cell adhesion molecules. It has been validated that CDH1 is regulated by miR-9, and another study has shown that expression of CDH1 is downmodulated after ROS exposure [39, 40]. The forkhead box O protein 1 (FOXO1) is a tumor suppressor implied in prostate cancer and the insulin signaling pathway. It has been validated that FOXO1 is downregulated by miR-9, miR-27, miR-96, miR-153, miR-182, miR-183, and miR-186. On the other hand, two other studies showed induction of FOXO expression upon oxidative stress [41–43].

Also of note, one of our predicted common targets of oxidative stress-modulated miRNAs (Table 1), namely, CBL, is the component of three of the KEGG pathways identified (the insulin, T cell receptor, and ErbB signaling pathways).

For a full list of all KEGG pathways common to ≥2 of the 13 oxidative stress-modulated miRNAs, see Supplementary Table 4.

### 3.4. New Candidate MicroRNAs Potentially Involved in Oxidative Stress Responses

We inserted the common gene targets from Table 1 into the TargetScan database [13–15] to export the miRNAs modulating each of them. Using SID1.0 [17] to find the common miRNAs, we identified new candidate miRNAs that may be involved in oxidative stress responses. As shown in Table 3 and Supplementary Table 5, miR-9 was found to be common to all the 13 targets analyzed, while the 26 other miRNAs indicated in the tables were common to 9, 10, 11, or 12 of them.

Six of the identified miRNAs have already been described to be modulated during oxidative stress responses: miR-9, miR-16, miR-29b, miR-128, miR-144, and miR-200c (highlighted in italics in Table 3 and bold in Supplementary Table 5). The other 21 miRNAs that our analysis identified have not yet been ascribed a direct role in oxidative stress, and they are therefore novel candidate oxidative stress response-related miRNAs. Biological functions of several of these miRNAs have been reported. miR-101 has been the object of many studies and is well known to be involved in Akt signaling and the MAPK pathway. Moreover, miR-101 has been described to be related to various cancers and to target various tumor-suppressor genes and oncogenes, as well as to be involved in cell proliferation, migration, invasion, angiogenesis, and cell death. miR-429, miR-15, miR-195, miR-93, and miR-497 have been implicated in carcinogenesis. miR-124 is a well-studied miRNA, which seems to function as a tumor suppressor and to be implicated in cell differentiation (amongst others closely linked to neuronal differentiation) as well and in regulation of the cytoskeleton. miR-17 seems to be implied in leukemia and lung cancer. It suppresses apoptosis and regulates MAP14. miR-106b targets PTEN and has a prooncogenic function when overexpressed, by also suppressing Bim and p21 expression. miR-519 reduces cell proliferation. miR-27a is involved in cell cycle regulation and is linked with leukemia and carcinogenesis; its downregulation inhibits cell proliferation. miR-23b is involved in carcinogenesis and cell migration, and miR-23a regulates cardiac hypertrophy. miR-424 promotes angiogenesis. Of note, a study by Li et al. screened for miRNAs altered during stress-induced premature cell senescence (which may be related to oxidative stress) and identified several of the miRNAs that are in our list as novel candidate oxidative stress response-related miRNAs (miR-15a/b, miR-106a/b, miR-20a, and miR-195) [44].

Interestingly, by using the TargetScan prediction tool, we found that Nrf2 could be modulated by several of the miRNAs that we have predicted to be involved in oxidative stress responses (Table 3), namely, miR-128, miR-144, miR-548n, miR-101, miR-23a/b, miR-27a/b, miR-106a/b, miR-17, miR-20a/b, and miR-93.

For a full list of all miRNAs that target ≥2 of the 13 gene targets of oxidative stress-modulated miRNAs, see Supplementary Table 6.

**Figure 2:**
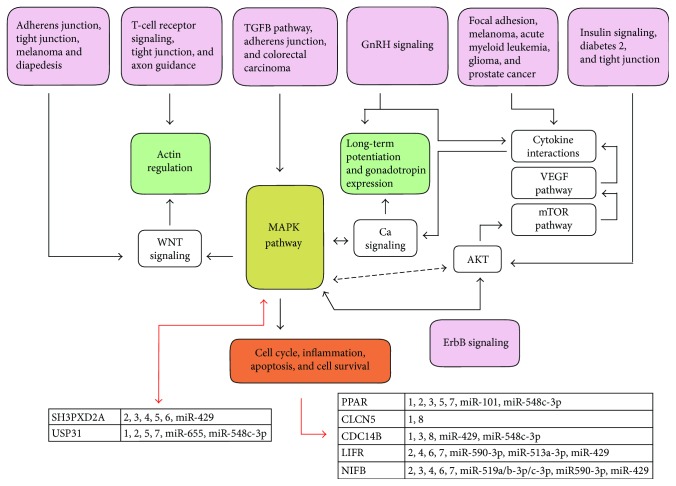
MicroRNAs predicted to target genes involved in the pathways modulated by oxidative stress. We added to the previous simplified flow diagram (shown in Figure 1) the miRNAs that we predicted to be putative novel actors in oxidative stress responses (shown in Table 3) and grouped them according to their common gene targets and overall relationship to cellular pathways/responses. 1 = miR-195, miR-424, miR-15a/b, and miR-497; 2 = miR-106a/b, miR-17, miR-20a/b, miR-93, and miR-519d; 3 = miR-124 and miR-506; 4 = miR-655, miR-548c-3p, and miR-101; 5 = miR-519a/b-3p/c-3p, miR-590-3p, and miR-513a-3p; 6 = miR-548n, miR-23a/b, and miR-27a/b; 7 = miR-548p and miR-429; and 8 = miR-548n and miR-27a/b.

Taken together, all these pieces of information add new and interesting suggestions on the miRNA-dependent regulation of gene networks and cellular processes related to the oxidative stress response. This can be used to generate novel and testable hypotheses, as well as in the planning of further levels of experimental investigation. The bioinformatics approach shown in this paper takes into account the complexity of the regulatory interactome of individual miRNAs with the range of target genes involved in cellular pathways, comparing at the same time different miRNA hubs and regulatory subnetworks (Figure 2) during the biological/functional interpretation of the retrieved information, which is further discussed in the next section.

### 3.5. Biological and Functional Interpretation of Oxidative Stress-Associated MicroRNAs

**Figure 3:**
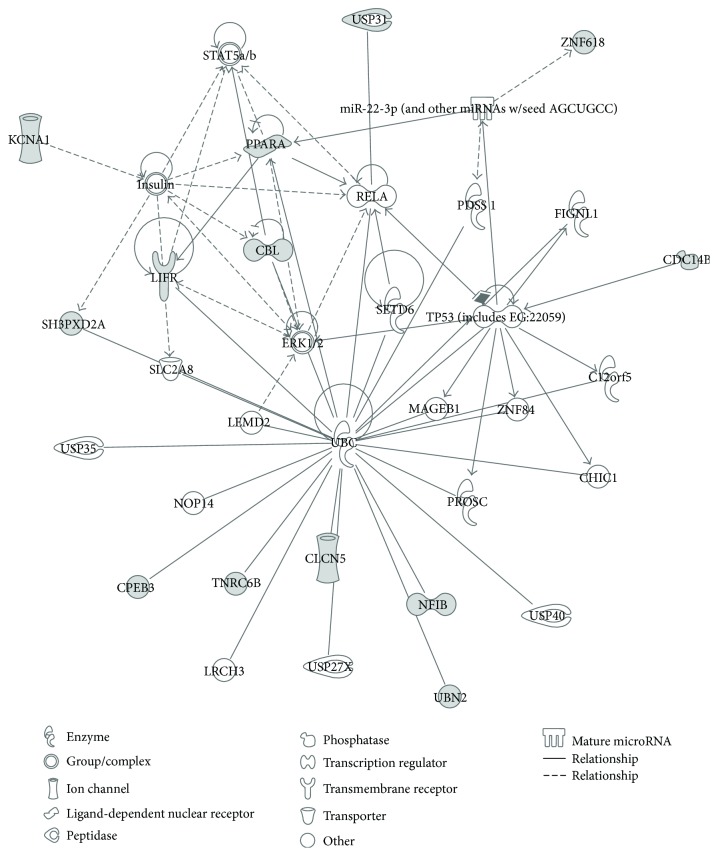
Graphical representation of the molecular relationships between oxidative stress-modulated miRNA gene targets. Indirect interactions exist between the protein products of the 13 gene targets of oxidative stress-modulated miRNAs predicted from our SID1.0 analysis (shown in Table 1). Molecules are represented as nodes, and the biological relationship between two nodes is represented as an edge (line). The dotted edges indicate indirect interactions whereas the others indicate direct interactions. All edges are supported by at least 1 reference from the literature, from a textbook, or from canonical information stored in the Ingenuity Pathways Knowledge Base. Nodes are displayed using various shapes that represent the functional class of the gene product, as indicated in the legend. The filled grey nodes indicate the 13 target molecules obtained from our SID1.0 analysis.

**Figure 4:**
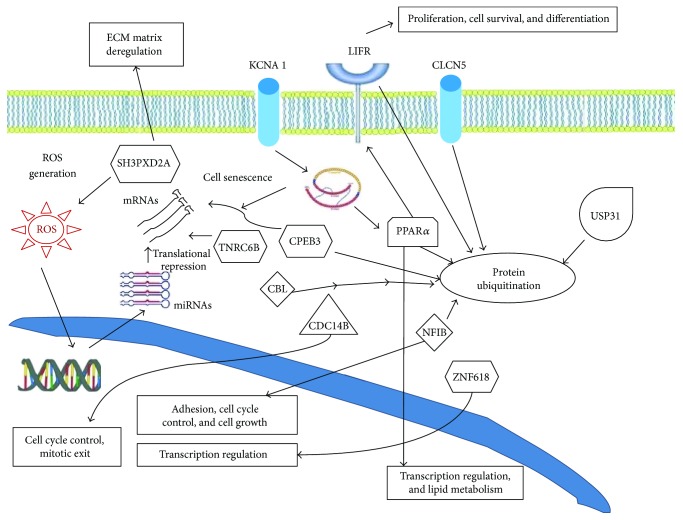
Simplified overview of some of the effects that our identified oxidative stress-modulated miRNA gene targets may generate and their relation to protein ubiquitination.

The Ingenuity Pathways Analysis (IPA; Ingenuity Systems, http://www.ingenuity.com) is a useful resource to perform a functional analysis of gene targets identified during laboratory or in silico investigations, providing biological interpretations of complex events or matrices of data, which is the case in the exploration of cellular networks of molecular and functional interactions. In our case, the gene targets of miRNAs involved in oxidative stress identified by SID1.0 analysis were explored with this web tool to identify possible biological functions (Figures 3 and 4).

The resulting network indicates that the predicted gene targets of miRNAs involved in oxidative stress are associated with a few discrete common pathways. Firstly and most importantly, almost all targets are connected and flow in the “protein ubiquitination pathway.” The protein ubiquitination pathway is implied in the degradation of regulatory proteins involved in a variety of cellular processes, such as cell cycle control, cell proliferation, apoptosis, DNA repair, transcriptional regulation, cell surface receptors, ion channel regulation, and antigen presentation. In fact, the IPA analysis of our data associated the obtained results with the network of cell-cell communication mechanisms, cell cycle regulation, and cellular development. The main targets in relation to these cellular responses, NFIB [45], LIFR [46], PPAR*α* [47], and CBEP3 [48], are important regulatory proteins, which may undergo modulation effects by the ubiquitination pathway. The other top molecular and cellular functions in our functional analysis suggested possible effects of oxidative stress on cellular morphology, cellular assembly and organization, cellular function and maintenance, cellular movement, and energy production.

Our analyses predict that oxidative stress can lead to miRNA-mediated deregulated expression of three membrane proteins: CLCN5, KCNA1, and LIFR (Figure 4). The first two are ion transporters/channels. CLCN5 is an antiport system for chloride and protons, which is important for endosome acidification and renal tubular function [49]. KCNA1 is a voltage-gated potassium channel engaged in cell communication in the brain [50]. LIFR is a membrane protein and polyfunctional cytokine that affects cell differentiation, survival, and proliferation [46]. The transcriptional factors PPAR*α* and NFIB are engaged in lipid metabolism (PPAR*α*) and adhesion, cell cycle control, and cell growth (NFIB). The function of ZNF618 is unknown, but it is also associated with transcriptional regulation. The dual specificity protein tyrosine phosphatase CDC14B is involved in cell cycle control and mitotic exit due to dephosphorylation of the tumor suppressor protein p53, which is further involved in the oxidative DNA damage response [51]. Interestingly, some of the identified gene targets, such as TNRC6B and CBEP3, may participate in the functional modulation of mRNAs, thus pointing to discrete effects in the miRNA-induced translational repression of groups of genes that are likely to respond to conditions of cellular oxidative stress. In more detail, TNRC6B is itself an essential component for the translational repression mediated by miRNAs and siRNAs. TNRC6B is recruited to miRNA targets through an interaction between its N-terminal domain and an Argonaute protein; TNRC6B then promotes translational repression and/or degradation of miRNA targets through a C-terminal silencing domain [52]. CBEP3 is a RNA-binding protein that represses translation of its target mRNAs and negatively regulates EGFR signaling in neurons.

SH3PXDA2A is an adapter protein involved in invadopodia, podosome formation, extracellular matrix degradation, and invasiveness of some cancer cells. It binds matrix metalloproteinases, NADPH oxidases (NOX), and phosphoinositides and acts as an organizer protein that allows NOX1- or NOX3-dependent ROS generation and cellular localization [53].

Finally, the protein ubiquitination pathway seems to be targeted by oxidative stress-modulated miRNAs at different levels. On the one hand, 6 of the 13 identified miRNA targets (PPAR*α*, LIFR, CLCN5, NFIB, CBEP3, and SH3PXD2A) are known to be ubiquitin C substrates, whereas on the other hand CBL and USP31 are members of the ubiquitination pathway itself. USP31 is involved in recognition and processing of polyubiquitin precursors as well as of ubiquitinated proteins. CBL, a RING finger E3 ubiquitin ligase, is required to convey substrates to proteasomal degradation, mediating the transfer of ubiquitin from ubiquitin-conjugating enzymes to specific substrates; at the same time, it is known to be a negative regulator of many signal transduction pathways [54].

Intriguingly, many of the miRNAs shown in Table 3 have been demonstrated to be able to target enzymes involved in the ubiquitination process. miR-9 targets CBL (E3 ubiquitin ligase) [55]. miR-16 and miR-128 downregulate translation of the Smurf2 protein, a tumor-suppressive ubiquitin ligase [56]. miR-101 targets MARCH7, a member of the RING finger protein family of E3 ubiquitin ligases. miR-429 and miR-200 are involved in the expression of various ULM (ubiquitin-like modifiers) proteins [57]. miR-497 is a negative regulator SMURF1 (SMAD-specific E3 ubiquitin protein ligase 1) [58], and miR-17 negatively regulates TNF-*α* signaling by acting on the modulation of the protein ubiquitin processes [59]. miR-93 directly suppresses ubiquitin ligase b-TRCP2 expression [60], and miR-124 was found to directly influence the activity of ubiquitin-specific proteases (USP) 2 and 14 [61, 62].

### 3.6. The Predicted Oxidative Stress-Modulated miRNAs That Affect the Protein Ubiquitination Pathway Also Regulate Autophagy

Autophagy dysfunction has been observed in various human pathologies such as cancer, autoimmune and infectious diseases, and neurodegenerative disorders. Ischemia and oxidative stress cause protein aggregation and mitochondrial dysfunction. On one side, upstream processes such as protein misfolding and aggregation lead to autophagy induction, whereas on the other side autophagy may fail if protein aggregation is very extreme.

Oxidative damage can irreversibly modify proteins, so that they need to be degraded. Two proteolytic systems execute the degradation task: the ubiquitin-proteasome system (UPS) and the autophagic-lysosomal system. The proteasome needs to unfold and linearize proteins in order to do its job (since the polypeptides need to be threaded through the very narrow core of the proteasome), and therefore the UPS cannot degrade irreversibly misfolded or aggregated proteins. The autophagic process, on the other hand, can degrade proteins of any size or form, since the material that is to be degraded is collected into double- or multimembrane vacuoles termed “autophagosomes,” which are very large compared to proteins, and often can have a diameter of 500–1500 nm. Thus, autophagy can degrade not only macromolecules but also even large organelles, including mitochondria. Interestingly, ubiquitin is used as a “degrade me” recognition signal not only in the ubiquitin-proteasome system but also in many forms of autophagy. The underlying mechanistic principle for this lies in ubiquitin-binding subtypes of the so-called autophagy receptors (e.g., p62/SQSMT1, NBR1, OPTN, TOLLIP, Cue5, NDP52, and TAX1BP1). These autophagy receptors have one protein domain that binds to ubiquitinated cargo and another domain that interacts with a class of small proteins that are attached to the autophagic membrane (Atg8 family proteins). Thereby, the cargo is recruited to the forming autophagosome (the “phagophore”) and eventually sequestered inside it after the phagophore has closed. Upon fusion of the autophagosome with lysosomes, the inner autophagosomal membrane and the sequestered material are degraded by lysosomal enzymes, and the building blocks are recycled into the cytoplasm for reuse by the cell. Unlike the UPS, which only degrades ubiquitinated proteins, the ubiquitin “degrade me” signal is used to initiate the autophagic degradation of a variety of cellular structures, including mitochondria, RNA granules, protein aggregates, bacteria, the midbody, and even proteasomes [63]. Damaged mitochondria can be ubiquitinated at multiple outer mitochondrial membrane proteins and recognized by the autophagy receptors p62/SQSMT1, OPTN, NDP52, and TAX1BP1, whereas ubiquitinated protein aggregates can be targeted for autophagic degradation via binding to the autophagy receptors p62/SQSMT1, NBR1, OPTN, TOLLIP, and Cue5 [63]. In sum, ubiquitin-dependent autophagy can serve an important role in helping cells to neutralize damage caused by oxidative stress.

Given the established role of ubiquitin in autophagy, and the fact that our integrated in silico analysis suggested that the effects of oxidative stress-related miRNAs intersect at the protein ubiquitination pathway, we reasoned that these miRNAs may also converge to regulate autophagy. Indeed, through a literature search, we found that in fact all the oxidative stress-related miRNAs that we had identified by the reverse approach shown in Table 3 and that target enzymes involved in the ubiquitination process (miR-9, miR-16, miR-17, miR-93, miR-101, miR-124, miR-128, miR-200, miR-429, and miR-497) have been reported to regulate autophagy [64–76]. For example, miR-9 affects autophagy by targeting the products of the essential autophagy-related (ATG) genes ATG5 [68] and Beclin1 [75], whereas miR-17 targets ATG7 [66], Beclin1 [64], and p62/SQSTM1 [70]. miR-124 targets Beclin1 [74] and p62/SQSTM1 [71]. Modulation of this set of miRNAs that we identified by in silico analysis (miR-9, miR-16, miR-17, miR-93, miR-101, miR-124, miR-128, miR-200, miR-429, and miR-497) thus appears to have a strong potential to concertedly mediate the effects of oxidative stress on both protein ubiquitination and autophagy. This illustrates how our in silico approach can lead to specific predictions and hypotheses that can be further tested experimentally.

### 3.7. Additional Links between Oxidative Stress-Modulated miRNAs and Autophagy

Autophagy, including selective autophagy, can in many cases operate independently of ubiquitination [63]. We therefore also performed literature searches for links between autophagy and the other candidates from our list of predicted oxidative stress-modulated miRNAs shown in Table 3. We found that several of these miRNAs have been implicated in autophagy regulation, that is, miR-15a, miR-20a/b, miR-23a/b, miR-29a, miR-106a/b, miR-195, and miR-590-3p [67, 70, 73, 77–81]. For example, miR-29a targets the product of the essential autophagy-related gene ATG9A as well as that of the master transcriptional regulator of lysosomal biogenesis and autophagy, TFEB [77]. As another example, miR-195 has been shown to target GABARAPL1 (of the Atg8 family) [78] and ATG14 [79].

Finally, we examined if we could identify links between autophagy and gene products from our predicted set of common gene targets of oxidative stress-modulated miRNAs shown in Table 1. We found that at least two of the target genes have been firmly demonstrated to play a role in autophagy, namely, CBL and PPAR*α*. Interestingly, CBL can act as an autophagy receptor to deliver Src (a nonreceptor tyrosine kinase) and paxillin (an adapter protein of focal adhesion complexes) for autophagosomal degradation [82, 83]. Of note, CBL performs this function independently of its E3 ligase activity [82, 83]. PPAR*α* has been demonstrated to control the transcription of a large number of autophagy-related genes in starved hepatocytes [84]. Moreover, fenofibrate, which is a potent agonist of PPAR*α* that often also induces ROS, was shown to induce autophagy-dependent degradation of KEAP1, which again led to increased activity of Nrf2 and protection against oxidative stress damage [85].

Altogether, these identified links indicate that oxidative stress-modulated miRNAs may affect autophagy in a broad sense (i.e., both ubiquitin-dependent and ubiquitin-independent autophagy), and given the important role of autophagy in the oxidative stress response, this warrants further in silico and experimental studies.

## 4. Conclusions

In the present paper, we demonstrate the potential of using in silico approaches as a starting point to address the complex challenge of understanding the integrated biological roles of microRNAs in oxidative stress responses. By using existing results from published experimental data, combined with databases and software, we predicted a set of 13 common gene targets and 25 commonly affected cellular pathways from a set of 13 miRNAs whose expression levels have been reported to be modulated by oxidative stress. Furthermore, from the 13 identified gene targets, we predicted 21 novel candidate oxidative stress-related miRNAs. Ingenuity pathway analyses of our 13 identified target genes indicated main interaction networks and biological impacts of their gene products and importantly led us to identify protein ubiquitination as a dominating pathway commonly affected by this set of gene targets of oxidative stress-modulated miRNAs. Finally, we found by literature search that we could also draw several lines of connections between our identified novel candidate oxidative stress-related miRNAs and the autophagic pathway.

We consider that our study has two main values. Firstly, the data we have generated can be used as a starting point and resource for the generation of testable hypotheses and further experimental research to address specific questions related to the role of miRNAs in oxidative stress-mediated biological responses. Secondly, the study has value as a proof of principle of how in silico analyses can be used to make advances from already existing data in the field of miRNAs and oxidative stress. Indeed, as the experimental data on oxidative stress-modulated miRNAs, their gene targets, and their biological effects are continuously increasing, there will be more and more to gain by utilizing types of in silico approaches like the ones applied in the present paper. Our study shows that we already have come to the point where such analyses can provide meaningful and useful output, which otherwise would be very hard to reach. We therefore envision an important role for this line of research to be constantly evolving and integrated with biological experimental work, to accelerate our advances in the understanding of the oxidative stress response.

## Figures and Tables

**Table 1:** Common gene targets of microRNAs with possible role in oxidative stress. Common targets of 13 oxidative stress-modulated miRNAs: hsa-let7f (91 elements), hsa-miR-9 (936 elements), hsa-miR-16 (294 elements), hsa-miR-21 (105 elements), hsa-miR-22 (330 elements), hsa-miR-29b (158 elements), hsa-miR-99a (24 elements), hsa-miR-125b (412 elements), hsa-miR-128 (785 elements), hsa-miR-143 (263 elements), hsa-miR-144 (647 elements), hsa-miR-155 (281 elements), and hsa-miR-200c (34 elements). Listed are 13 gene targets found to be common to 5, 6, or 7 of the 13 oxidative stress-modulated miRNAs. The database used for this analysis was TargetScan [13]. The miRNA-target genes marked in italics have already been validated and described to be involved in oxidative stress responses.

Target gene	Annotation	Common miRNAs
ZNF618	Zinc finger protein 618	hsa-miR-9; hsa-miR-22; hsa-miR-125b; hsa-miR-128; hsa-miR-143; hsa-miR-144; hsa-miR-155
SH3PXD2A	SH3 and PX domains 2A	hsa-miR-9; hsa-miR-22; hsa-miR-29b; hsa-miR-143; hsa-miR-144; hsa-miR-155
TNRC6B	Trinucleotide repeat containing 6B	hsa-miR-9; hsa-miR-16; hsa-miR-29b; hsa-miR-128; hsa-miR-144; hsa-miR-22
CBL	Cas-Br-M (murine) ecotropic retroviral transforming sequence	let-7f; hsa-miR-9; hsa-miR-22; hsa-miR-143; hsa-miR-155
CPEB3	Cytoplasmic polyadenylation element binding protein 3	hsa-miR-9; hsa-miR-16; hsa-miR-21; hsa-miR-128; hsa-miR-144
*PPARA*	Peroxisome proliferator-activated receptor alpha	hsa-miR-9; hsa-miR-22; hsa-miR-21; hsa-miR-128; hsa-miR-144
CLCN5	Chloride channel 5 (nephrolithiasis 2, X-linked, Dent disease)	hsa-miR-9; hsa-miR-16; hsa-miR-22; hsa-miR-128; hsa-miR-155
*CDC14B*	CDC14 cell division cycle 14 homolog B (*S. cerevisiae*)	hsa-miR-9; hsa-miR-16; hsa-miR-125b; hsa-miR-128; hsa-miR-144
LIFR	Leukemia inhibitory factor receptor alpha	hsa-miR-9; hsa-miR-143; hsa-miR-21; hsa-miR-128; hsa-miR-144
KCNA1	Potassium voltage-gated channel, shaker-related subfamily, member 1 (episodic ataxia with myokymia)	hsa-miR-9; hsa-miR-155; hsa-miR-21; hsa-miR-128; hsa-miR-144
USP31	Ubiquitin-specific peptidase 31	hsa-miR-9; hsa-miR-16; hsa-miR-155; hsa-miR-200c; hsa-miR-144
tcag7.1228	Hypothetical protein FLJ25778	hsa-miR-9; hsa-miR-16; hsa-miR-21; hsa-miR-128; hsa-miR-144
*NFIB*	Nuclear factor I/B	hsa-miR-9; hsa-miR-22; hsa-miR-21; hsa-miR-128; hsa-miR-29b

Note: see Supplementary Table 1 for this table in Excel format, and see Supplementary Table 2 for a full list of gene targets found to be common to ≥2 of the 13 oxidative stress-modulated miRNAs (i.e., all possible combinations).

**Table 2:** Common pathways (KEGG) of microRNAs associated with oxidative stress. Common pathways (KEGG pathway IDs) of hsa-let7f (129 elements), hsa-miR-9 (140 elements), hsa-miR-16 (117 elements), hsa-miR-21 (64 elements), hsa-miR-22 (101 elements), hsa-miR-29b (126 elements), hsa-miR-99a (40 elements), hsa-miR-125b (126 elements), hsa-miR-128 (103 elements), hsa-miR-143 (80 elements), hsa-miR-144 (110 elements), hsa-miR-155 (70 elements), and hsa-miR-200c (113 elements). The pathways are common to all the 13 oxidative stress-modulated miRNAs. The KEGG pathway name (first column), the gene symbols involved in each pathway (second column), and the ID of the pathway used by the KEGG database (third column) are indicated. The database used for this analysis was DIANA-MicroT 3.0 [3].

KEGG pathway name	Gene symbol	KEGG pathway ID
MAPK signaling pathway	FGF12, PRKCA, MAP3K1, RPS6KA4, SOS1, MAP3K3, SRF, PAK2, MAP2K7, FGF18, RAP1B, MAPKAPK2, ACVR1B, TGFBR2, FGF5, DUSP6, ACVR1C, PDGFRB	hsa04010
Melanoma	FGF12, FGF18, FGF5, CDH1, IGF1, PDGFRB, PIK3R3, PDGFC	hsa05218
Colorectal cancer	RALGDS, SOS1, TCF7, ACVR1B, TGFBR2, ACVR1C, DCC, PDGFRB, PIK3R3, SMAD4	hsa05210
Glioma	PRKCA, SHC2, SOS1, SHC1, IGF1, PDGFRB, PIK3R3	hsa05214
Adherens junction	CTNNA1, ACTN2, VCL, TCF7, BAIAP2, SRC, ACVR1B, SSX2IP, TGFBR2, ACVR1C, CDH1, SMAD4	hsa04520
Focal adhesion	ITGB4, PRKCA, SHC2, ACTN2, TNC, DIAPH1, VCL, COL5A1, ITGA6, SOS1, VAV3, SHC1, SRC, PAK4, PAK2, PAK6, THBS2, RAP1B, IGF1, PDGFRB, PIK3R3, PDGFC	hsa04510
TGF-beta signaling pathway	ID4, SMURF1, INHBB, THBS2, ACVR1B, TGFBR2, ACVR1C, SMAD4	hsa04350
mTOR signaling pathway	TSC1, ULK2, IGF1, PIK3R3, EIF4E	hsa04150
Prostate cancer	CCNE2, SOS1, TCF7, FOXO1, CREB5, IGF1, PDGFRB, PIK3R3, PDGFC, CREB3L2	hsa05215
Wnt signaling pathway	WNT4, PRKCA, TCF7, VANGL1, PSEN1, PPARD, SMAD4	hsa04310
Cytokine-cytokine receptor interaction	LEP, CNTFR, LIFR, CXCL11, INHBB, KITLG, TNFRSF21, ACVR1B, TGFBR2, PDGFRB, PDGFC	hsa04060
Basal cell carcinoma	WNT4, TCF7, PTCH1	hsa05217
Type II diabetes mellitus	SOCS4, PIK3R3	hsa04930
Cell adhesion molecules (CAMs)	SDC2, CLDN14, SDC1, NFASC, ITGA6, NEO1, ALCAM, NLGN2, CDH1	hsa04514
Regulation of actin cytoskeleton	ITGB4, MYH9, FGF12, ACTN2, DIAPH1, VCL, ARPC1A, ARHGEF7, PIP4K2B, ITGA6, SOS1, VAV3, BAIAP2, DIAPH2, PAK4, PAK2, PAK6, FGF18, FGF5, SLC9A1, PDGFRB, PIK3R3	hsa04810
Long-term potentiation	PRKCA, RAP1B	hsa04720
Insulin signaling pathway	TSC1, SHC2, SOCS4, CBL, SOS1, FOXO1, SHC1, PIK3R3, EIF4E	hsa04910
Leukocyte transendothelial migration	CTNNA1, PRKCA, ACTN2, CLDN14, VCL, VAV3, RAP1B, PIK3R3	hsa04670
Tight junction	CTNNA1, MYH9, PPP2R4, PRKCA, ACTN2, CLDN14, AMOTL1, SRC, CSDA	hsa04530
Axon guidance	SRGAP3, PLXNA2, NTNG1, EPHB2, SEMA6D, EPHA7, NRP1, PAK4, PAK2, PAK6, DCC, EPHB4, EFNA1	hsa04360
Calcium signaling pathway	PRKCA, SLC8A1, ADCY9, PDGFRB	hsa04020
T cell receptor signaling pathway	CBL, SOS1, VAV3, PAK4, PAK2, PAK6, PIK3R3	hsa04660
GnRH signaling pathway	PRKCA, MAP3K1, SOS1, MAP3K3, SRC, ADCY9, MAP2K7	hsa04912
ErbB signaling pathway	PRKCA, SHC2, CBL, SOS1, SHC1, SRC, ABL2, PAK4, PAK2, PAK6, MAP2K7, PIK3R3	hsa04012
Acute myeloid leukemia	SOS1, TCF7, JUP, PPARD, PIK3R3	hsa05221
VEGF signaling pathway	PRKCA, SHC2, SRC, MAPKAPK2, PIK3R3	hsa04370

Note: see Supplementary Table 3 for this table in Excel format, and see Supplementary Table 4 for a full list of all KEGG pathways common to ≥2 of the 13 oxidative stress-modulated miRNAs.

**Table 3:** miRNAs predicted to be involved in oxidative stress responses. Identification of miRNAs predicted to simultaneously target the genes identified in Table 1: ZNF618 (114 elements), SH3PXD2A (135 elements), TNRC6B (329 elements), CBL (740 elements), CPEB3 (178 elements), PPARA (933 elements), CLCN5 (167 elements), CDC14B (64 elements), LIFR (553 elements), KCNA1 (549 elements), USP31 (93 elements), tcag7.1228 (210 elements), and NFIB (281 elements). Shown are miRNAs (column 1) common to ≥9 of the 13 gene targets with the corresponding annotation. We found one miRNA (hsa-miR-9) common to all 13 gene targets. The database used for this analysis was TargetScan [13]. The miRNAs marked in italics have already been described to be involved in oxidative stress response.

Common miRNAs	Target genes
*hsa-miR-9*	CBL; CPEB3; PPARA; CLCN5; CDC14B; LIFR; KCNA1; USP31; ZNF618; tcag7.1228; NFIB; SH3PXD2A; TNRC6B
hsa-miR-548c-3p	CBL; CPEB3; PPARA; CDC14B; LIFR; KCNA1; USP31; ZNF618; tcag7.1228; NFIB; SH3PXD2A; TNRC6B
*hsa-miR-128* *hsa-miR-144* hsa-miR-548n	CBL; CPEB3; PPARA; CLCN5; CDC14B; LIFR; KCNA1; ZNF618; tcag7.1228; NFIB; TNRC6B CBL; CPEB3; PPARA; CDC14B; LIFR; KCNA1; USP31; ZNF618; tcag7.1228; SH3PXD2A; TNRC6B CPEB3; PPARA; CLCN5; CDC14B; LIFR; KCNA1; USP31; ZNF618; NFIB; SH3PXD2A; TNRC6B
hsa-miR-655 hsa-miR-548p hsa-miR-101 *hsa-miR-29*a/*b*/c	CBL; CPEB3; LIFR; KCNA1; USP31; ZNF618; tcag7.1228; NFIB; SH3PXD2A; TNRC6B CBL; CPEB3; PPARA; LIFR; KCNA1; USP31; ZNF618; tcag7.1228; NFIB; TNRC6B CBL; CPEB3; PPARA; LIFR; KCNA1; ZNF618; tcag7.1228; NFIB; SH3PXD2A; TNRC6B CBL; CPEB3; PPARA; CLCN5; LIFR; ZNF618; tcag7.1228; NFIB; SH3PXD2A; TNRC6B
hsa-miR-195 *hsa-miR-16* hsa-miR-424 hsa-miR-15a/b hsa-miR-497 hsa-miR-23a/b hsa-miR-27a/b hsa-miR-519a/b-3p/c-3p hsa-miR-106a/b hsa-miR-17 hsa-miR-20a/b hsa-miR-93 hsa-miR-590-3p hsa-miR-124 hsa-miR-506 hsa-miR-513a-3p hsa-miR-429 *hsa-miR-200*b/*c*	CBL; CPEB3; PPARA; CLCN5; CDC14B; KCNA1; USP31; tcag7.1228; TNRC6B CBL; CPEB3; PPARA; CLCN5; CDC14B; KCNA1; USP31; tcag7.1228; TNRC6B CBL; CPEB3; PPARA; CLCN5; CDC14B; KCNA1; USP31; tcag7.1228; TNRC6B CBL; CPEB3; PPARA; CLCN5; CDC14B; KCNA1; USP31; tcag7.1228; TNRC6B CBL; CPEB3; PPARA; CLCN5; CDC14B; KCNA1; USP31; tcag7.1228; TNRC6B CBL; PPARA; LIFR; KCNA1; USP31; tcag7.1228; NFIB; SH3PXD2A; TNRC6B CPEB3; PPARA; CLCN5; CDC14B; LIFR; KCNA1; USP31; NFIB; TNRC6B CPEB3; PPARA; KCNA1; USP31; ZNF618; tcag7.1228; NFIB; SH3PXD2A; TNRC6B CPEB3; PPARA; LIFR; KCNA1; USP31; ZNF618; NFIB; SH3PXD2A; TNRC6B CPEB3; PPARA; LIFR; KCNA1; USP31; ZNF618; NFIB; SH3PXD2A; TNRC6B CPEB3; PPARA; LIFR; KCNA1; USP31; ZNF618; NFIB; SH3PXD2A; TNRC6B CPEB3; PPARA; LIFR; KCNA1; USP31; ZNF618; NFIB; SH3PXD2A; TNRC6B PPARA; LIFR; KCNA1; USP31; ZNF618; tcag7.1228; NFIB; SH3PXD2A; TNRC6B CBL; PPARA; CDC14B; KCNA1; ZNF618; tcag7.1228; NFIB; SH3PXD2A; TNRC6B CBL; PPARA; CDC14B; KCNA1; ZNF618; tcag7.1228; NFIB; SH3PXD2A; TNRC6B CBL; PPARA; LIFR; KCNA1; USP31; ZNF618; tcag7.1228; SH3PXD2A; TNRC6B CBL; CDC14B; LIFR; KCNA1; USP31; tcag7.1228; NFIB; SH3PXD2A; TNRC6B CBL; PPARA; CDC14B; LIFR; KCNA1; USP31; tcag7.1228; NFIB; SH3PXD2A

Note: see Supplementary Table 5 for this table in Excel format, and see Supplementary Table 6 for a full list of miRNAs that target ≥2 of the 13 gene targets.

## Supplementary Materials

Supplementary 1 Table 1: common gene targets of microRNAs with possible roles in oxidative stress. This table is an Excel format of Table 1.

Supplementary 2 Table 2: common gene targets of microRNAs with possible roles in oxidative stress. This table relates to Table 1 and Supplementary Table 1, where gene targets found to be common to 5, 6, or 7 of the 13 oxidative stress-modulated miRNAs were shown. Here, we show the full list of gene targets found to be common to ≥2 of the 13 oxidative stress-modulated miRNAs (i.e., all possible combinations): hsa-let7f (91 elements), hsa-miR-9 (936 elements), hsa-miR-16 (294 elements), hsa-miR-21 (105 elements), hsa-miR-22 (330 elements), hsa-miR-29b (158 elements), hsa-miR-99a (24 elements), hsa-miR-125b (412 elements), hsa-miR-128 (785 elements), hsa-miR-143 (263 elements), hsa-miR-144 (647 elements), hsa-miR-155 (281 elements), and hsa-miR-200c (34 elements). Raw SID1.0 data list is shown. Line numbers indicate code positioning.

Supplementary 3 Table 3: common pathways (KEGG pathway IDs) of microRNAs associated with oxidative stress. This table is an Excel format of Table 2.

Supplementary 4 Table 4: common pathways (KEGG pathway IDs) of microRNAs associated with oxidative stress. This table relates to Table 2 and Supplementary Table 3, where pathways common to all 13 oxidative stress-modulated miRNAs were shown. Here, we show the full list of all KEGG pathways common to ≥2 of the 13 oxidative stress-modulated miRNAs.

Supplementary 5 Table 5: common pathways (KEGG pathway IDs) of microRNAs associated with oxidative stress. This table is an Excel format of Table 3.

Supplementary 6 Table 6: miRNAs predicted to be involved in oxidative stress responses. This table relates to Table 3 and Supplementary Table 5, where miRNAs that target ≥9 of the 13 gene targets of oxidative stress-modulated miRNAs (listed in Table 1) were shown. Here, we show the full list of miRNAs that target ≥2 of the 13 gene targets of oxidative stress-modulated miRNAs (from SID1.0 raw data list).
