# Supplementary material for: From Oxidative Stress Damage to Pathways, Networks, and Autophagy via MicroRNAs
Source: Oxid Med Cell Longev. 2018 Apr 12;2018:4968321. doi: 10.1155/2018/4968321 (PMC5932428; doi:10.1155/2018/4968321)
Supplement: Supplementary 2 — Table 2: common gene targets of microRNAs with possible roles in oxidative stress. This table relates to Table 1 and Supplementary Table 1, where gene targets found to be common to 5, 6, or 7 of the 13 oxidative stress-modulated miRNAs were shown. Here, we show the full list of gene targets found to be common to ≥2 of the 13 oxidative stress-modulated miRNAs (i.e., all possible combinations): hsa-let7f (91 elements), hsa-miR-9 (936 elements), hsa-miR-16 (294 elements), hsa-miR-21 (105 elements), hsa-miR-22 (330 elements), hsa-miR-29b (158 elements), hsa-miR-99a (24 elements), hsa-miR-125b (412 elements), hsa-miR-128 (785 elements), hsa-miR-143 (263 elements), hsa-miR-144 (647 elements), hsa-miR-155 (281 elements), and hsa-miR-200c (34 elements). Raw SID1.0 data list is shown. Line numbers indicate code positioning. [file 4968321.f2.pdf]

Suppl. Table 2: Common gene targets of microRNAs with possible role in oxidative stress.

This Table relates to Table 1 and Suppl. Table 1, where gene targets found to be common to 7, 6, or 5 of the 13 oxidative stress-modulated miRNAs were shown. Here we show the full list of gene targets found to be common to ≥ 2 of the 13 oxidative stress-modulated miRNAs (i.e. all possible combinations); hsa-let7f (91 elements), hsa-miR-9 (936 elements), hsa-miR-16 (294 elements), hsa-miR-21 (105 elements), hsa-miR-22 (330 elements), hsa-miR-29b (158 elements), hsa-miR-99a (24 elements), hsa-miR-125b (412 elements), hsa-miR-128 (785 elements), hsa-miR-143 (263 elements), hsa-miR-144 (647 elements), hsa-miR-155 (281 elements) and hsa-miR-200c (34 elements). Raw SID1.0 data list is shown. Line numbers indicate code positioning.

The original dataset is: dataset_58.txt

----------------------- Files in dataset
----

1 /	5801-let7f.txt	91	elements
2 /	5802-mir-9.txt	936	elements
3 /	5803-mir-16.txt	294	elements
4 /	5804-miR-21.txt	105	elements
5 /	5805-mir-22.txt	330	elements
6 /	5806-mir-29b.txt	158	elements
7 /	5807-mir-99a.txt	24	elements
8 /	5808-mir-125b.txt	412	elements
9 /	5809-mir-128.txt	785	elements
10 /	5810-mir-143.txt	263	elements
11 /	5811-mir-144.txt	647	elements
12 /	5812-mir-155.txt	281	elements
13 /	5813-mir-200c.txt	34	elements

----------------------- Files in dataset

***** Index of miRNA Codes *****

SMARCAD1 5801-let7f.txt
line 1
SMARCAD1 5812-mir-155.txt
line 270

BZW1 5801-let7f.txt
line 5
BZW1 5811-mir-144.txt
line 424

ZNF583 5801-let7f.txt
line 7
ZNF583 5810-mir-143.txt
line 14

FNDC3A 5801-let7f.txt
line 8

FNDC3A 5811-mir-144.txt
line 15

HIF1AN 5801-let7f.txt
line 11
HIF1AN 5805-mir-22.txt
line 185

DVL3 5801-let7f.txt
line 15
DVL3 5808-mir-125b.txt
line 374

FZD4 5801-let7f.txt
line 19
FZD4 5811-mir-144.txt
line 192

PTAR1 5801-let7f.txt
line 20
PTAR1 5802-mir-9.txt
line 114
PTAR1 5808-mir-125b.txt
line 145
PTAR1 5812-mir-155.txt
line 70

MAN2A2 5801-let7f.txt
line 24
MAN2A2 5809-mir-128.txt
line 343

MIB1 5801-let7f.txt
line 26
MIB1 5803-mir-16.txt
line 60
MIB1 5808-mir-125b.txt
line 216

CCR7 5801-let7f.txt
line 30
CCR7 5804-miR-21.txt
line 55

SLC2A12 5801-let7f.txt
line 39
SLC2A12 5812-mir-155.txt
line 76

ZBTB5 5801-let7f.txt
line 41
ZBTB5 5806-mir-29b.txt
line 15
ZBTB5 5811-mir-144.txt
line 484

SNX16 5801-let7f.txt
line 42
SNX16 5803-mir-16.txt
line 106

PRKAA2 5801-let7f.txt
line 43
PRKAA2 5808-mir-125b.txt
line 138

PXDN 5801-let7f.txt
line 45
PXDN 5802-mir-9.txt
line 52

SPRYD4 5801-let7f.txt
line 49
SPRYD4 5802-mir-9.txt
line 840

TMED5 5801-let7f.txt
line 50
TMED5 5809-mir-128.txt
line 643
TMED5 5811-mir-144.txt
line 336

SCYL3 5801-let7f.txt
line 52
SCYL3 5802-mir-9.txt
line 313

GREB1 5801-let7f.txt
line 56
GREB1 5802-mir-9.txt
line 475

CACNG4 5801-let7f.txt
line 58
CACNG4 5806-mir-29b.txt
line 144

PPP1R12B 5801-let7f.txt
line 59
PPP1R12B 5808-mir-125b.txt
line 33

NTRK3 5801-let7f.txt
line 62
NTRK3 5809-mir-128.txt
line 499

DTX4 5801-let7f.txt
line 66
DTX4 5808-mir-125b.txt
line 282
DTX4 5809-mir-128.txt
line 175

EIF2C4 5801-let7f.txt
line 67
EIF2C4 5812-mir-155.txt
line 103

SYT1 5801-let7f.txt
line 68
SYT1 5809-mir-128.txt
line 79

IGF1 5801-let7f.txt
line 70
IGF1 5802-mir-9.txt
line 892
IGF1 5803-mir-16.txt
line 281
IGF1 5809-mir-128.txt

line 741

KTELC1 5801-let7f.txt
line 71
KTELC1 5809-mir-128.txt
line 22

CBL 5801-let7f.txt
line 76
CBL 5802-mir-9.txt
line 853
CBL 5805-mir-22.txt
line 3
CBL 5810-mir-143.txt
line 165
CBL 5812-mir-155.txt
line 108

TIMM17B 5801-let7f.txt
line 80
TIMM17B 5808-mir-125b.txt
line 284

GRIK2 5801-let7f.txt
line 81
GRIK2 5811-mir-144.txt
line 278

LCOR 5801-let7f.txt
line 82
LCOR 5811-mir-144.txt
line 467

BCL7A 5801-let7f.txt
line 83
BCL7A 5804-miR-21.txt
line 39

CALM1 5801-let7f.txt
line 84
CALM1 5810-mir-143.txt
line 210

CCNJL 5801-let7f.txt

line 85
CCNJL 5805-mir-22.txt
line 20

MGA 5801-let7f.txt
line 86
MGA 5802-mir-9.txt
line 531

ARID3A 5801-let7f.txt
line 87
ARID3A 5808-mir-125b.txt
line 293

ICMT 5801-let7f.txt
line 89
ICMT 5802-mir-9.txt
line 422
ICMT 5807-mir-99a.txt
line 22

FBXL19 5801-let7f.txt
line 91
FBXL19 5802-mir-9.txt
line 922
FBXL19 5805-mir-22.txt
line 174

ONECUT2 5802-mir-9.txt
line 1
ONECUT2 5808-mir-125b.txt
line 345
ONECUT2 5809-mir-128.txt
line 39
ONECUT2 5811-mir-144.txt
line 39

FBN1 5802-mir-9.txt
line 4
FBN1 5811-mir-144.txt
line 44

ENTPD1 5802-mir-9.txt
line 6

ENTPD1 5808-mir-125b.txt
line 23

SHROOM4 5802-mir-9.txt
line 8
SHROOM4 5806-mir-29b.txt
line 108

GABRB2 5802-mir-9.txt
line 13
GABRB2 5804-miR-21.txt
line 21
GABRB2 5811-mir-144.txt
line 601

PRRX1 5802-mir-9.txt
line 20
PRRX1 5811-mir-144.txt
line 207

PRDM1 5802-mir-9.txt
line 21
PRDM1 5808-mir-125b.txt
line 76

SFXN2 5802-mir-9.txt
line 23
SFXN2 5809-mir-128.txt
line 18

CNOT6L 5802-mir-9.txt
line 24
CNOT6L 5803-mir-16.txt
line 29
CNOT6L 5805-mir-22.txt
line 155
CNOT6L 5811-mir-144.txt
line 406

XPO4 5802-mir-9.txt
line 25
XPO4 5803-mir-16.txt
line 288

STARD13	5802-mir-9.txt
line 28	
STARD13	5808-mir-125b.txt
line 1	
NID2	5802-mir-9.txt
line 30	
NID2	5811-mir-144.txt
line 385	
SOCS4	5802-mir-9.txt
line 31	
SOCS4	5808-mir-125b.txt
line 248	
MTHFD2	5802-mir-9.txt
line 33	
MTHFD2	5805-mir-22.txt
line 200	
MGC13057	5802-mir-9.txt
line 37	
MGC13057	5808-mir-125b.txt
line 358	
KLF5	5802-mir-9.txt
line 41	
KLF5	5804-miR-21.txt
line 40	
KLF5	5810-mir-143.txt
line 106	
ITGA6	5802-mir-9.txt
line 45	
ITGA6	5806-mir-29b.txt
line 129	
ITGA6	5810-mir-143.txt
line 91	
AP1S3	5802-mir-9.txt
line 47	
AP1S3	5811-mir-144.txt
line 30	

MTMR2 5802-mir-9.txt
line 49
MTMR2 5805-mir-22.txt
line 133
MTMR2 5811-mir-144.txt
line 581

KIAA1553 5802-mir-9.txt
line 53
KIAA1553 5809-mir-128.txt
line 519
KIAA1553 5811-mir-144.txt
line 368

CPEB2 5802-mir-9.txt
line 55
CPEB2 5803-mir-16.txt
line 11
CPEB2 5810-mir-143.txt
line 237
CPEB2 5811-mir-144.txt
line 347

KLHL18 5802-mir-9.txt
line 59
KLHL18 5809-mir-128.txt
line 750

C10orf119 5802-mir-9.txt
line 62
C10orf119 5806-mir-29b.txt
line 66

FOXP1 5802-mir-9.txt
line 65
FOXP1 5805-mir-22.txt
line 217
FOXP1 5811-mir-144.txt
line 560

NEDD4 5802-mir-9.txt
line 67
NEDD4 5809-mir-128.txt
line 436

VAV3 5802-mir-9.txt
line 71
VAV3 5808-mir-125b.txt
line 354
VAV3 5809-mir-128.txt
line 487
VAV3 5812-mir-155.txt
line 33

RBMS3 5802-mir-9.txt
line 72
RBMS3 5804-miR-21.txt
line 87
RBMS3 5812-mir-155.txt
line 16

NUS1 5802-mir-9.txt
line 75
NUS1 5809-mir-128.txt
line 246

CCNG1 5802-mir-9.txt
line 79
CCNG1 5809-mir-128.txt
line 483

TGFBI 5802-mir-9.txt
line 81
TGFBI 5804-miR-21.txt
line 18

MESDC1 5802-mir-9.txt
line 83
MESDC1 5809-mir-128.txt
line 744

FBN2 5802-mir-9.txt
line 84
FBN2 5805-mir-22.txt
line 269
FBN2 5811-mir-144.txt
line 49

DLGAP2 5802-mir-9.txt
line 86
DLGAP2 5803-mir-16.txt
line 226
DLGAP2 5805-mir-22.txt
line 275
DLGAP2 5806-mir-29b.txt
line 60

LEP 5802-mir-9.txt
line 91
LEP 5809-mir-128.txt
line 728

COL15A1 5802-mir-9.txt
line 93
COL15A1 5806-mir-29b.txt
line 26

ProSAPiP1 5802-mir-9.txt
line 94
ProSAPiP1 5809-mir-128.txt
line 764

NR5A2 5802-mir-9.txt
line 100
NR5A2 5809-mir-128.txt
line 659

ADAMTS3 5802-mir-9.txt
line 106
ADAMTS3 5811-mir-144.txt
line 79

HMGA2 5802-mir-9.txt
line 110
HMGA2 5803-mir-16.txt
line 167

RBM9 5802-mir-9.txt
line 115
RBM9 5805-mir-22.txt
line 151

C1orf173 5802-mir-9.txt
line 119
C1orf173 5809-mir-128.txt
line 409

ZBTB41 5802-mir-9.txt
line 120
ZBTB41 5803-mir-16.txt
line 222
ZBTB41 5812-mir-155.txt
line 11

EPHA7 5802-mir-9.txt
line 124
EPHA7 5811-mir-144.txt
line 346

CPEB3 5802-mir-9.txt
line 126
CPEB3 5803-mir-16.txt
line 68
CPEB3 5804-miR-21.txt
line 7
CPEB3 5809-mir-128.txt
line 665
CPEB3 5811-mir-144.txt
line 411

SLC5A3 5802-mir-9.txt
line 129
SLC5A3 5809-mir-128.txt
line 261

TTYH2 5802-mir-9.txt
line 130
TTYH2 5811-mir-144.txt
line 341

PCGF5 5802-mir-9.txt
line 132
PCGF5 5805-mir-22.txt
line 150

MDGA1 5802-mir-9.txt

line	138	
MDGA1		5809-mir-128.txt
line	777	
KCNMB2		5802-mir-9.txt
line	140	
KCNMB2		5811-mir-144.txt
line	298	
VANGL1		5802-mir-9.txt
line	142	
VANGL1		5809-mir-128.txt
line	37	
VANGL1		5811-mir-144.txt
line	5	
MUM1L1		5802-mir-9.txt
line	146	
MUM1L1		5805-mir-22.txt
line	7	
SIX4		5802-mir-9.txt
line	149	
SIX4		5810-mir-143.txt
line	55	
SIX4		5811-mir-144.txt
line	334	
ZC3H12A		5802-mir-9.txt
line	150	
ZC3H12A		5811-mir-144.txt
line	322	
SYNJ1		5802-mir-9.txt
line	151	
SYNJ1		5803-mir-16.txt
line	48	
TOMM20		5802-mir-9.txt
line	160	
TOMM20		5803-mir-16.txt
line	265	
TOMM20		5812-mir-155.txt
line	126	

GLS 5802-mir-9.txt
line 164
GLS 5808-mir-125b.txt
line 226

BACE1 5802-mir-9.txt
line 167
BACE1 5806-mir-29b.txt
line 141
BACE1 5809-mir-128.txt
line 580

HUNK 5802-mir-9.txt
line 171
HUNK 5805-mir-22.txt
line 137
HUNK 5809-mir-128.txt
line 456

PPARA 5802-mir-9.txt
line 178
PPARA 5804-miR-21.txt
line 34
PPARA 5805-mir-22.txt
line 296
PPARA 5809-mir-128.txt
line 640
PPARA 5811-mir-144.txt
line 258

SIRT1 5802-mir-9.txt
line 179
SIRT1 5805-mir-22.txt
line 43
SIRT1 5809-mir-128.txt
line 402
SIRT1 5812-mir-155.txt
line 224

FOXG1 5802-mir-9.txt
line 182
FOXG1 5811-mir-144.txt
line 494

ID4 5802-mir-9.txt
line 184
ID4 5811-mir-144.txt
line 226

FOXP4 5802-mir-9.txt
line 185
FOXP4 5809-mir-128.txt
line 692

ATP11B 5802-mir-9.txt
line 186
ATP11B 5809-mir-128.txt
line 324

FIGNL2 5802-mir-9.txt
line 187
FIGNL2 5803-mir-16.txt
line 294

RNF111 5802-mir-9.txt
line 189
RNF111 5803-mir-16.txt
line 207

SMARCD2 5802-mir-9.txt
line 190
SMARCD2 5808-mir-125b.txt
line 340
SMARCD2 5810-mir-143.txt
line 250

UBASH3B 5802-mir-9.txt
line 197
UBASH3B 5808-mir-125b.txt
line 295

ASXL1 5802-mir-9.txt
line 202
ASXL1 5812-mir-155.txt
line 257

SLC9A1 5802-mir-9.txt

```
line   205
SLC9A1                     5805-mir-22.txt
line   302

SHROOM2                    5802-mir-9.txt
line   208
SHROOM2                    5806-mir-29b.txt
line    30

ADAMTS6                    5802-mir-9.txt
line   209
ADAMTS6                    5806-mir-29b.txt
line    22

PHF8                       5802-mir-9.txt
line   210
PHF8                       5805-mir-22.txt
line    55

CLCN5                      5802-mir-9.txt
line   214
CLCN5                      5803-mir-16.txt
line    24
CLCN5                      5805-mir-22.txt
line   279
CLCN5                      5809-mir-128.txt
line   301
CLCN5                      5812-mir-155.txt
line   143

LSM14A                     5802-mir-9.txt
line   219
LSM14A                     5811-mir-144.txt
line   157
LSM14A                     5812-mir-155.txt
line    83

CCNE2                      5802-mir-9.txt
line   221
CCNE2                      5811-mir-144.txt
line   281

CCDC6                      5802-mir-9.txt
line   224
```

CCDC6 5809-mir-128.txt
line 730

PIGZ 5802-mir-9.txt
line 226
PIGZ 5809-mir-128.txt
line 302

ENAH 5802-mir-9.txt
line 228
ENAH 5809-mir-128.txt
line 157
ENAH 5811-mir-144.txt
line 592

ADAMTS9 5802-mir-9.txt
line 230
ADAMTS9 5806-mir-29b.txt
line 12

GMEB2 5802-mir-9.txt
line 234
GMEB2 5811-mir-144.txt
line 363

S100PBP 5802-mir-9.txt
line 235
S100PBP 5812-mir-155.txt
line 100

LIN28 5802-mir-9.txt
line 236
LIN28 5808-mir-125b.txt
line 137
LIN28 5809-mir-128.txt
line 385

ANK2 5802-mir-9.txt
line 238
ANK2 5811-mir-144.txt
line 297

TULP4 5802-mir-9.txt
line 241

TULP4 5811-mir-144.txt
line 330

BAHD1 5802-mir-9.txt
line 244
BAHD1 5806-mir-29b.txt
line 150
BAHD1 5809-mir-128.txt
line 605

ANKRD13A 5802-mir-9.txt
line 245
ANKRD13A 5805-mir-22.txt
line 82

ZNF395 5802-mir-9.txt
line 247
ZNF395 5808-mir-125b.txt
line 400

SNRK 5802-mir-9.txt
line 249
SNRK 5803-mir-16.txt
line 103
SNRK 5805-mir-22.txt
line 186

SLC20A2 5802-mir-9.txt
line 250
SLC20A2 5803-mir-16.txt
line 34
SLC20A2 5811-mir-144.txt
line 23

C5orf41 5802-mir-9.txt
line 252
C5orf41 5809-mir-128.txt
line 557
C5orf41 5811-mir-144.txt
line 365

CCDC88A 5802-mir-9.txt
line 256
CCDC88A 5809-mir-128.txt

line 469
CCDC88A 5811-mir-144.txt
line 331

OTUD3 5802-mir-9.txt
line 258
OTUD3 5811-mir-144.txt
line 524

DIXDC1 5802-mir-9.txt
line 261
DIXDC1 5811-mir-144.txt
line 470

ITPKC 5802-mir-9.txt
line 262
ITPKC 5809-mir-128.txt
line 515

CNTN3 5802-mir-9.txt
line 264
CNTN3 5811-mir-144.txt
line 203

ARID1A 5802-mir-9.txt
line 267
ARID1A 5811-mir-144.txt
line 36

RAB34 5802-mir-9.txt
line 270
RAB34 5812-mir-155.txt
line 216

ZIC5 5802-mir-9.txt
line 271
ZIC5 5806-mir-29b.txt
line 110

ERG 5802-mir-9.txt
line 272
ERG 5809-mir-128.txt
line 706

RAPH1	5802-mir-9.txt
line 273	
RAPH1	5812-mir-155.txt
line 93	
FBXL3	5802-mir-9.txt
line 275	
FBXL3	5811-mir-144.txt
line 97	
BCL2L11	5802-mir-9.txt
line 276	
BCL2L11	5811-mir-144.txt
line 535	
TMEM196	5802-mir-9.txt
line 277	
TMEM196	5811-mir-144.txt
line 150	
PBRM1	5802-mir-9.txt
line 278	
PBRM1	5811-mir-144.txt
line 356	
POU2F3	5802-mir-9.txt
line 279	
POU2F3	5809-mir-128.txt
line 229	
KITLG	5802-mir-9.txt
line 280	
KITLG	5809-mir-128.txt
line 340	
KITLG	5811-mir-144.txt
line 154	
SPG20	5802-mir-9.txt
line 281	
SPG20	5804-miR-21.txt
line 75	
CPEB4	5802-mir-9.txt
line 283	

CPEB4 5809-mir-128.txt
line 675

HDAC5 5802-mir-9.txt
line 285
HDAC5 5809-mir-128.txt
line 288

FRMD4A 5802-mir-9.txt
line 294
FRMD4A 5809-mir-128.txt
line 663

ROD1 5802-mir-9.txt
line 298
ROD1 5810-mir-143.txt
line 205
ROD1 5811-mir-144.txt
line 500

TGFBR2 5802-mir-9.txt
line 306
TGFBR2 5804-miR-21.txt
line 86
TGFBR2 5811-mir-144.txt
line 566
TGFBR2 5812-mir-155.txt
line 260

CEP350 5802-mir-9.txt
line 307
CEP350 5803-mir-16.txt
line 292
CEP350 5811-mir-144.txt
line 619

TMCC1 5802-mir-9.txt
line 309
TMCC1 5809-mir-128.txt
line 507
TMCC1 5811-mir-144.txt
line 482

HK2 5802-mir-9.txt

line   310
HK2   5810-mir-143.txt
line   23

SH2B3   5802-mir-9.txt
line   311
SH2B3   5808-mir-125b.txt
line   346
SH2B3   5811-mir-144.txt
line   441

NPTX1   5802-mir-9.txt
line   312
NPTX1   5809-mir-128.txt
line   708
NPTX1   5811-mir-144.txt
line   78

TRIM2   5802-mir-9.txt
line   318
TRIM2   5812-mir-155.txt
line   212

RTF1   5802-mir-9.txt
line   322
RTF1   5803-mir-16.txt
line   218

KIAA0240   5802-mir-9.txt
line   324
KIAA0240   5811-mir-144.txt
line   316

BCL11A   5802-mir-9.txt
line   325
BCL11A   5806-mir-29b.txt
line   61
BCL11A   5811-mir-144.txt
line   458

DHX40   5802-mir-9.txt
line   327
DHX40   5812-mir-155.txt
line   58

ANKH 5802-mir-9.txt
line 329
ANKH 5808-mir-125b.txt
line 336

MKL2 5802-mir-9.txt
line 332
MKL2 5810-mir-143.txt
line 131
MKL2 5811-mir-144.txt
line 10

SGK269 5802-mir-9.txt
line 333
SGK269 5806-mir-29b.txt
line 139
SGK269 5809-mir-128.txt
line 754

STK38L 5802-mir-9.txt
line 334
STK38L 5806-mir-29b.txt
line 86
STK38L 5809-mir-128.txt
line 578

FAM46A 5802-mir-9.txt
line 335
FAM46A 5804-miR-21.txt
line 15
FAM46A 5808-mir-125b.txt
line 288
FAM46A 5811-mir-144.txt
line 111

UNKL 5802-mir-9.txt
line 339
UNKL 5806-mir-29b.txt
line 137
UNKL 5809-mir-128.txt
line 448

ST8SIA4 5802-mir-9.txt

line 340
ST8SIA4 5808-mir-125b.txt
line 148
ST8SIA4 5810-mir-143.txt
line 207

SSX2IP 5802-mir-9.txt
line 341
SSX2IP 5811-mir-144.txt
line 55

DYRK2 5802-mir-9.txt
line 344
DYRK2 5811-mir-144.txt
line 93

KIAA0831 5802-mir-9.txt
line 346
KIAA0831 5803-mir-16.txt
line 31

GRIK3 5802-mir-9.txt
line 348
GRIK3 5809-mir-128.txt
line 44

RNF144A 5802-mir-9.txt
line 350
RNF144A 5808-mir-125b.txt
line 375
RNF144A 5809-mir-128.txt
line 83

FURIN 5802-mir-9.txt
line 351
FURIN 5805-mir-22.txt
line 276
FURIN 5808-mir-125b.txt
line 322
FURIN 5809-mir-128.txt
line 703

TLK1 5802-mir-9.txt
line 352

TLK1 5803-mir-16.txt
line 9

ACVR1C 5802-mir-9.txt
line 354
ACVR1C 5809-mir-128.txt
line 590

CAMKK2 5802-mir-9.txt
line 360
CAMKK2 5811-mir-144.txt
line 328

CCNJ 5802-mir-9.txt
line 361
CCNJ 5808-mir-125b.txt
line 29
CCNJ 5809-mir-128.txt
line 619

MAP3K1 5802-mir-9.txt
line 365
MAP3K1 5804-miR-21.txt
line 33

AMMECR1 5802-mir-9.txt
line 368
AMMECR1 5810-mir-143.txt
line 224
AMMECR1 5811-mir-144.txt
line 515

SFRS1 5802-mir-9.txt
line 370
SFRS1 5809-mir-128.txt
line 500

PSD3 5802-mir-9.txt
line 371
PSD3 5810-mir-143.txt
line 136

FOXP2 5802-mir-9.txt
line 372

FOXP2 5809-mir-128.txt
line 404

IKZF2 5802-mir-9.txt
line 373
IKZF2 5809-mir-128.txt
line 600

FAM117A 5802-mir-9.txt
line 376
FAM117A 5805-mir-22.txt
line 270
FAM117A 5810-mir-143.txt
line 176

FAM118A 5802-mir-9.txt
line 377
FAM118A 5808-mir-125b.txt
line 136

C14orf147 5802-mir-9.txt
line 378
C14orf147 5812-mir-155.txt
line 139

CPSF6 5802-mir-9.txt
line 382
CPSF6 5808-mir-125b.txt
line 78

PCDH10 5802-mir-9.txt
line 383
PCDH10 5811-mir-144.txt
line 572

TNKS 5802-mir-9.txt
line 384
TNKS 5804-miR-21.txt
line 60

GLDN 5802-mir-9.txt
line 387
GLDN 5805-mir-22.txt
line 207

CLCN4 5802-mir-9.txt
line 388
CLCN4 5803-mir-16.txt
line 70

CDC14B 5802-mir-9.txt
line 389
CDC14B 5803-mir-16.txt
line 89
CDC14B 5808-mir-125b.txt
line 198
CDC14B 5809-mir-128.txt
line 624
CDC14B 5811-mir-144.txt
line 184

CNNM1 5802-mir-9.txt
line 390
CNNM1 5808-mir-125b.txt
line 202
CNNM1 5810-mir-143.txt
line 246

SNF1LK 5802-mir-9.txt
line 392
SNF1LK 5809-mir-128.txt
line 645

TXNDC5 5802-mir-9.txt
line 393
TXNDC5 5809-mir-128.txt
line 338

RNF169 5802-mir-9.txt
line 403
RNF169 5806-mir-29b.txt
line 149

SOS1 5802-mir-9.txt
line 407
SOS1 5803-mir-16.txt
line 233
SOS1 5809-mir-128.txt

line 158
SOS1 5812-mir-155.txt
line 201

SLC19A2 5802-mir-9.txt
line 408
SLC19A2 5811-mir-144.txt
line 492

ZBTB39 5802-mir-9.txt
line 412
ZBTB39 5803-mir-16.txt
line 63
ZBTB39 5805-mir-22.txt
line 34
ZBTB39 5809-mir-128.txt
line 381

XRN1 5802-mir-9.txt
line 414
XRN1 5812-mir-155.txt
line 84

MAP7 5802-mir-9.txt
line 416
MAP7 5803-mir-16.txt
line 97

RHOBTB1 5802-mir-9.txt
line 417
RHOBTB1 5806-mir-29b.txt
line 51
RHOBTB1 5812-mir-155.txt
line 178

ARID3B 5802-mir-9.txt
line 431
ARID3B 5805-mir-22.txt
line 172
ARID3B 5808-mir-125b.txt
line 18
ARID3B 5810-mir-143.txt
line 109

RAB11FIP4	5802-mir-9.txt
line 435	
RAB11FIP4	5811-mir-144.txt
line 459	
EDEM3	5802-mir-9.txt
line 437	
EDEM3	5809-mir-128.txt
line 537	
ABI1	5802-mir-9.txt
line 442	
ABI1	5811-mir-144.txt
line 40	
SLC6A6	5802-mir-9.txt
line 444	
SLC6A6	5809-mir-128.txt
line 505	
STAC	5802-mir-9.txt
line 445	
STAC	5809-mir-128.txt
line 599	
STAC	5810-mir-143.txt
line 36	
STAC	5811-mir-144.txt
line 435	
FLRT3	5802-mir-9.txt
line 448	
FLRT3	5809-mir-128.txt
line 481	
FLRT3	5811-mir-144.txt
line 230	
KIAA1045	5802-mir-9.txt
line 451	
KIAA1045	5809-mir-128.txt
line 573	
BCL6	5802-mir-9.txt
line 452	
BCL6	5811-mir-144.txt

line 289

PANK3 5802-mir-9.txt
line 453
PANK3 5804-miR-21.txt
line 92
PANK3 5811-mir-144.txt
line 232

ZBTB34 5802-mir-9.txt
line 455
ZBTB34 5808-mir-125b.txt
line 360
ZBTB34 5809-mir-128.txt
line 432
ZBTB34 5811-mir-144.txt
line 211

MAPKAPK2 5802-mir-9.txt
line 457
MAPKAPK2 5808-mir-125b.txt
line 387

THAP2 5802-mir-9.txt
line 458
THAP2 5807-mir-99a.txt
line 1
THAP2 5809-mir-128.txt
line 146

EIF5A2 5802-mir-9.txt
line 469
EIF5A2 5803-mir-16.txt
line 280
EIF5A2 5808-mir-125b.txt
line 281

KLHDC5 5802-mir-9.txt
line 470
KLHDC5 5804-miR-21.txt
line 35
KLHDC5 5805-mir-22.txt
line 211

COL5A1	5802-mir-9.txt
line 471	
COL5A1	5809-mir-128.txt
line 335	
COL5A1	5810-mir-143.txt
line 148	
AFAP1	5802-mir-9.txt
line 476	
AFAP1	5809-mir-128.txt
line 628	
AFAP1	5811-mir-144.txt
line 373	
RPS6KA4	5802-mir-9.txt
line 477	
RPS6KA4	5809-mir-128.txt
line 781	
PDE7B	5802-mir-9.txt
line 478	
PDE7B	5809-mir-128.txt
line 192	
PDE7B	5811-mir-144.txt
line 404	
ATOH8	5802-mir-9.txt
line 482	
ATOH8	5808-mir-125b.txt
line 325	
PHF20L1	5802-mir-9.txt
line 484	
PHF20L1	5811-mir-144.txt
line 621	
PURA	5802-mir-9.txt
line 487	
PURA	5811-mir-144.txt
line 128	
FOXN3	5802-mir-9.txt
line 489	
FOXN3	5809-mir-128.txt

line 503

TGOLN2 5802-mir-9.txt
line 496
TGOLN2 5808-mir-125b.txt
line 50
TGOLN2 5809-mir-128.txt
line 597

NOTCH2 5802-mir-9.txt
line 499
NOTCH2 5803-mir-16.txt
line 61

VCL 5802-mir-9.txt
line 500
VCL 5806-mir-29b.txt
line 148

KIF21A 5802-mir-9.txt
line 502
KIF21A 5803-mir-16.txt
line 25

TES 5802-mir-9.txt
line 503
TES 5811-mir-144.txt
line 412

RAB33B 5802-mir-9.txt
line 504
RAB33B 5809-mir-128.txt
line 109

ZNF248 5802-mir-9.txt
line 507
ZNF248 5812-mir-155.txt
line 132

KLF12 5802-mir-9.txt
line 508
KLF12 5804-miR-21.txt
line 42
KLF12 5811-mir-144.txt

line 296

NEBL 5802-mir-9.txt
line 513
NEBL 5806-mir-29b.txt
line 128

PIP4K2B 5802-mir-9.txt
line 515
PIP4K2B 5805-mir-22.txt
line 89
PIP4K2B 5808-mir-125b.txt
line 338

AEBP2 5802-mir-9.txt
line 517
AEBP2 5811-mir-144.txt
line 394

MAP2K7 5802-mir-9.txt
line 521
MAP2K7 5808-mir-125b.txt
line 146
MAP2K7 5809-mir-128.txt
line 297

CD47 5802-mir-9.txt
line 528
CD47 5812-mir-155.txt
line 120

HOXD1 5802-mir-9.txt
line 539
HOXD1 5808-mir-125b.txt
line 308

FXR1 5802-mir-9.txt
line 540
FXR1 5812-mir-155.txt
line 185

KIAA1217 5802-mir-9.txt
line 541
KIAA1217 5811-mir-144.txt

line 212

EN2 5802-mir-9.txt
line 543
EN2 5809-mir-128.txt
line 689
EN2 5812-mir-155.txt
line 82

FOXQ1 5802-mir-9.txt
line 548
FOXQ1 5809-mir-128.txt
line 318

ETS1 5802-mir-9.txt
line 551
ETS1 5811-mir-144.txt
line 159
ETS1 5812-mir-155.txt
line 42

GAB2 5802-mir-9.txt
line 553
GAB2 5808-mir-125b.txt
line 392

LPP 5802-mir-9.txt
line 554
LPP 5805-mir-22.txt
line 299
LPP 5808-mir-125b.txt
line 238

TRAM1 5802-mir-9.txt
line 560
TRAM1 5803-mir-16.txt
line 95

HRB 5802-mir-9.txt
line 561
HRB 5809-mir-128.txt
line 603
HRB 5811-mir-144.txt
line 543

PIK3C2A 5802-mir-9.txt
line 563
PIK3C2A 5811-mir-144.txt
line 260

GJC1 5802-mir-9.txt
line 564
GJC1 5808-mir-125b.txt
line 59

PHTF2 5802-mir-9.txt
line 566
PHTF2 5811-mir-144.txt
line 308
PHTF2 5813-mir-200c.txt
line 10

PALM2 5802-mir-9.txt
line 567
PALM2 5809-mir-128.txt
line 564
PALM2 5811-mir-144.txt
line 357
PALM2 5812-mir-155.txt
line 278

WAPAL 5802-mir-9.txt
line 568
WAPAL 5803-mir-16.txt
line 206

DYRK1A 5802-mir-9.txt
line 571
DYRK1A 5811-mir-144.txt
line 408

AAK1 5802-mir-9.txt
line 573
AAK1 5812-mir-155.txt
line 155

TMEM170B 5802-mir-9.txt
line 574

TMEM170B 5803-mir-16.txt
line 272
TMEM170B 5808-mir-125b.txt
line 133
TMEM170B 5809-mir-128.txt
line 437

SYT10 5802-mir-9.txt
line 575
SYT10 5803-mir-16.txt
line 39

BRUNOL6 5802-mir-9.txt
line 576
BRUNOL6 5806-mir-29b.txt
line 113

PLEKHA1 5802-mir-9.txt
line 578
PLEKHA1 5806-mir-29b.txt
line 88

RIPK5 5802-mir-9.txt
line 580
RIPK5 5811-mir-144.txt
line 585

YPEL2 5802-mir-9.txt
line 581
YPEL2 5809-mir-128.txt
line 376

RBM24 5802-mir-9.txt
line 582
RBM24 5803-mir-16.txt
line 102
RBM24 5810-mir-143.txt
line 57

WDTC1 5802-mir-9.txt
line 585
WDTC1 5805-mir-22.txt
line 289
WDTC1 5809-mir-128.txt

line 533

CREB5 5802-mir-9.txt
line 588
CREB5 5810-mir-143.txt
line 134

DCUN1D4 5802-mir-9.txt
line 590
DCUN1D4 5809-mir-128.txt
line 34
DCUN1D4 5811-mir-144.txt
line 416

C1orf144 5802-mir-9.txt
line 591
C1orf144 5805-mir-22.txt
line 321
C1orf144 5808-mir-125b.txt
line 224
C1orf144 5809-mir-128.txt
line 1

PJA2 5802-mir-9.txt
line 592
PJA2 5811-mir-144.txt
line 433

SON 5802-mir-9.txt
line 594
SON 5811-mir-144.txt
line 130

MBNL1 5802-mir-9.txt
line 596
MBNL1 5807-mir-99a.txt
line 16
MBNL1 5811-mir-144.txt
line 81

ADAMTS5 5802-mir-9.txt
line 597
ADAMTS5 5803-mir-16.txt
line 176

ADAMTS5 5805-mir-22.txt
line 298
ADAMTS5 5809-mir-128.txt
line 23

ZBTB38 5802-mir-9.txt
line 598
ZBTB38 5808-mir-125b.txt
line 355
ZBTB38 5811-mir-144.txt
line 109
ZBTB38 5812-mir-155.txt
line 6

DCP2 5802-mir-9.txt
line 600
DCP2 5809-mir-128.txt
line 346

AK3L1 5802-mir-9.txt
line 601
AK3L1 5803-mir-16.txt
line 85

ZBTB44 5802-mir-9.txt
line 602
ZBTB44 5803-mir-16.txt
line 21
ZBTB44 5810-mir-143.txt
line 22

MAF 5802-mir-9.txt
line 603
MAF 5808-mir-125b.txt
line 314
MAF 5810-mir-143.txt
line 122

SCN2B 5802-mir-9.txt
line 604
SCN2B 5808-mir-125b.txt
line 364
SCN2B 5810-mir-143.txt
line 138

CBFA2T2 5802-mir-9.txt
line 607
CBFA2T2 5811-mir-144.txt
line 315

ADAM11 5802-mir-9.txt
line 608
ADAM11 5805-mir-22.txt
line 248
ADAM11 5808-mir-125b.txt
line 315

RYBP 5802-mir-9.txt
line 611
RYBP 5809-mir-128.txt
line 46

FAM81A 5802-mir-9.txt
line 612
FAM81A 5803-mir-16.txt
line 72

AFF1 5802-mir-9.txt
line 613
AFF1 5810-mir-143.txt
line 19

PHF19 5802-mir-9.txt
line 618
PHF19 5803-mir-16.txt
line 7

HIC2 5802-mir-9.txt
line 619
HIC2 5808-mir-125b.txt
line 396

ATXN3 5802-mir-9.txt
line 623
ATXN3 5808-mir-125b.txt
line 241

C18orf25 5802-mir-9.txt

line 629
C18orf25 5809-mir-128.txt
line 775

NCOR2 5802-mir-9.txt
line 632
NCOR2 5803-mir-16.txt
line 203
NCOR2 5808-mir-125b.txt
line 240

ABL2 5802-mir-9.txt
line 634
ABL2 5808-mir-125b.txt
line 221
ABL2 5809-mir-128.txt
line 152
ABL2 5810-mir-143.txt
line 2

AK2 5802-mir-9.txt
line 635
AK2 5809-mir-128.txt
line 48

PLEKHA6 5802-mir-9.txt
line 636
PLEKHA6 5805-mir-22.txt
line 178

RC3H1 5802-mir-9.txt
line 641
RC3H1 5811-mir-144.txt
line 597

ANKRD12 5802-mir-9.txt
line 642
ANKRD12 5810-mir-143.txt
line 33

LARP1 5802-mir-9.txt
line 643
LARP1 5810-mir-143.txt
line 214

LIFR 5802-mir-9.txt
line 644
LIFR 5804-miR-21.txt
line 14
LIFR 5809-mir-128.txt
line 583
LIFR 5810-mir-143.txt
line 181
LIFR 5811-mir-144.txt
line 311

TNS1 5802-mir-9.txt
line 647
TNS1 5804-miR-21.txt
line 96

AMOTL1 5802-mir-9.txt
line 649
AMOTL1 5810-mir-143.txt
line 97

KIAA1549 5802-mir-9.txt
line 652
KIAA1549 5809-mir-128.txt
line 613

ANKRD52 5802-mir-9.txt
line 653
ANKRD52 5805-mir-22.txt
line 238
ANKRD52 5811-mir-144.txt
line 593

ACCN2 5802-mir-9.txt
line 654
ACCN2 5808-mir-125b.txt
line 290

CLCN6 5802-mir-9.txt
line 657
CLCN6 5809-mir-128.txt
line 227

PNRC2 5802-mir-9.txt
line 658
PNRC2 5803-mir-16.txt
line 205
PNRC2 5809-mir-128.txt
line 164

PTCH1 5802-mir-9.txt
line 660
PTCH1 5803-mir-16.txt
line 161
PTCH1 5809-mir-128.txt
line 607
PTCH1 5811-mir-144.txt
line 366

ARL4C 5802-mir-9.txt
line 662
ARL4C 5811-mir-144.txt
line 132

GRSF1 5802-mir-9.txt
line 666
GRSF1 5808-mir-125b.txt
line 299
GRSF1 5811-mir-144.txt
line 209

DNAJB1 5802-mir-9.txt
line 668
DNAJB1 5812-mir-155.txt
line 131

FAM63B 5802-mir-9.txt
line 669
FAM63B 5804-miR-21.txt
line 59

CTDSP2 5802-mir-9.txt
line 671
CTDSP2 5809-mir-128.txt
line 42

SLC1A1 5802-mir-9.txt

line 676
SLC1A1 5811-mir-144.txt
line 562

FMR1 5802-mir-9.txt
line 677
FMR1 5811-mir-144.txt
line 227

PLAG1 5802-mir-9.txt
line 678
PLAG1 5803-mir-16.txt
line 3
PLAG1 5809-mir-128.txt
line 433
PLAG1 5811-mir-144.txt
line 355

CNTFR 5802-mir-9.txt
line 679
CNTFR 5804-miR-21.txt
line 68

GABBR2 5802-mir-9.txt
line 680
GABBR2 5805-mir-22.txt
line 241
GABBR2 5809-mir-128.txt
line 457
GABBR2 5811-mir-144.txt
line 259

SHANK2 5802-mir-9.txt
line 684
SHANK2 5811-mir-144.txt
line 286
SHANK2 5812-mir-155.txt
line 227

MGAT1 5802-mir-9.txt
line 686
MGAT1 5809-mir-128.txt
line 697

SMURF1 5802-mir-9.txt
line 697
SMURF1 5803-mir-16.txt
line 22
SMURF1 5808-mir-125b.txt
line 169

C7orf42 5802-mir-9.txt
line 698
C7orf42 5809-mir-128.txt
line 755

EPAS1 5802-mir-9.txt
line 699
EPAS1 5812-mir-155.txt
line 279

SH3BP4 5802-mir-9.txt
line 700
SH3BP4 5808-mir-125b.txt
line 130

SMAD4 5802-mir-9.txt
line 706
SMAD4 5805-mir-22.txt
line 97
SMAD4 5808-mir-125b.txt
line 332
SMAD4 5811-mir-144.txt
line 468

C9orf5 5802-mir-9.txt
line 707
C9orf5 5803-mir-16.txt
line 197
C9orf5 5812-mir-155.txt
line 214

WSB1 5802-mir-9.txt
line 708
WSB1 5803-mir-16.txt
line 79
WSB1 5809-mir-128.txt
line 425

WSB1 5811-mir-144.txt
line 431

KCTD10 5802-mir-9.txt
line 709
KCTD10 5811-mir-144.txt
line 395

AATK 5802-mir-9.txt
line 710
AATK 5803-mir-16.txt
line 186

RCOR1 5802-mir-9.txt
line 711
RCOR1 5805-mir-22.txt
line 93
RCOR1 5812-mir-155.txt
line 72

SH3BGRL2 5802-mir-9.txt
line 712
SH3BGRL2 5809-mir-128.txt
line 116

SGK3 5802-mir-9.txt
line 714
SGK3 5811-mir-144.txt
line 65
SGK3 5812-mir-155.txt
line 51

GPATCH8 5802-mir-9.txt
line 715
GPATCH8 5808-mir-125b.txt
line 194

FAM155B 5802-mir-9.txt
line 716
FAM155B 5809-mir-128.txt
line 445

SEMA6D 5802-mir-9.txt
line 718

SEMA6D 5803-mir-16.txt
line 140
SEMA6D 5809-mir-128.txt
line 394

C2CD2 5802-mir-9.txt
line 719
C2CD2 5809-mir-128.txt
line 208
C2CD2 5811-mir-144.txt
line 623

RAI14 5802-mir-9.txt
line 721
RAI14 5809-mir-128.txt
line 674

TM9SF3 5802-mir-9.txt
line 722
TM9SF3 5811-mir-144.txt
line 570

ABCA1 5802-mir-9.txt
line 723
ABCA1 5809-mir-128.txt
line 139
ABCA1 5811-mir-144.txt
line 1

CREBZF 5802-mir-9.txt
line 726
CREBZF 5810-mir-143.txt
line 115

SGCD 5802-mir-9.txt
line 727
SGCD 5809-mir-128.txt
line 296

MAP3K7IP3 5802-mir-9.txt
line 728
MAP3K7IP3 5803-mir-16.txt
line 109
MAP3K7IP3 5809-mir-128.txt

line 536

RFXDC2 5802-mir-9.txt
line 729
RFXDC2 5806-mir-29b.txt
line 65

BNC2 5802-mir-9.txt
line 731
BNC2 5811-mir-144.txt
line 167
BNC2 5812-mir-155.txt
line 44

PITPNM2 5802-mir-9.txt
line 732
PITPNM2 5809-mir-128.txt
line 683

KCNA1 5802-mir-9.txt
line 736
KCNA1 5804-miR-21.txt
line 97
KCNA1 5809-mir-128.txt
line 253
KCNA1 5811-mir-144.txt
line 489
KCNA1 5812-mir-155.txt
line 265

CA7 5802-mir-9.txt
line 737
CA7 5809-mir-128.txt
line 417

HIPK2 5802-mir-9.txt
line 738
HIPK2 5809-mir-128.txt
line 604
HIPK2 5810-mir-143.txt
line 4
HIPK2 5811-mir-144.txt
line 321

GPAM 5802-mir-9.txt
line 741
GPAM 5809-mir-128.txt
line 9

FRY 5802-mir-9.txt
line 743
FRY 5803-mir-16.txt
line 271

ISL1 5802-mir-9.txt
line 744
ISL1 5809-mir-128.txt
line 108

ATP8A1 5802-mir-9.txt
line 752
ATP8A1 5805-mir-22.txt
line 59
ATP8A1 5809-mir-128.txt
line 51
ATP8A1 5811-mir-144.txt
line 563

EIF5 5802-mir-9.txt
line 753
EIF5 5809-mir-128.txt
line 399
EIF5 5811-mir-144.txt
line 284

UBR5 5802-mir-9.txt
line 756
UBR5 5809-mir-128.txt
line 67

RXRA 5802-mir-9.txt
line 757
RXRA 5809-mir-128.txt
line 700

ZFHX4 5802-mir-9.txt
line 759
ZFHX4 5809-mir-128.txt

line 648
ZFHX4 5811-mir-144.txt
line 87

CPEB1 5802-mir-9.txt
line 761
CPEB1 5805-mir-22.txt
line 95
CPEB1 5811-mir-144.txt
line 571

STX1A 5802-mir-9.txt
line 765
STX1A 5809-mir-128.txt
line 753

ELAVL2 5802-mir-9.txt
line 766
ELAVL2 5811-mir-144.txt
line 270

ALG9 5802-mir-9.txt
line 767
ALG9 5805-mir-22.txt
line 13
ALG9 5809-mir-128.txt
line 43

KIAA0247 5802-mir-9.txt
line 768
KIAA0247 5808-mir-125b.txt
line 379

IKZF4 5802-mir-9.txt
line 776
IKZF4 5805-mir-22.txt
line 12
IKZF4 5808-mir-125b.txt
line 141
IKZF4 5811-mir-144.txt
line 287

TFRC 5802-mir-9.txt
line 777

TFRC 5811-mir-144.txt
line 530

TEAD1 5802-mir-9.txt
line 778
TEAD1 5803-mir-16.txt
line 263
TEAD1 5809-mir-128.txt
line 89

RNF38 5802-mir-9.txt
line 780
RNF38 5809-mir-128.txt
line 180
RNF38 5811-mir-144.txt
line 558

FNDC3B 5802-mir-9.txt
line 782
FNDC3B 5808-mir-125b.txt
line 275
FNDC3B 5810-mir-143.txt
line 192

C17orf85 5802-mir-9.txt
line 785
C17orf85 5809-mir-128.txt
line 293

DICER1 5802-mir-9.txt
line 788
DICER1 5803-mir-16.txt
line 174
DICER1 5808-mir-125b.txt
line 276
DICER1 5811-mir-144.txt
line 325

USP31 5802-mir-9.txt
line 789
USP31 5803-mir-16.txt
line 162
USP31 5811-mir-144.txt
line 269

USP31 5812-mir-155.txt
line 184
USP31 5813-mir-200c.txt
line 12

ATP11C 5802-mir-9.txt
line 790
ATP11C 5810-mir-143.txt
line 34

DCBLD2 5802-mir-9.txt
line 794
DCBLD2 5809-mir-128.txt
line 733
DCBLD2 5811-mir-144.txt
line 105

RERE 5802-mir-9.txt
line 798
RERE 5806-mir-29b.txt
line 99
RERE 5809-mir-128.txt
line 543

ZNF618 5802-mir-9.txt
line 802
ZNF618 5805-mir-22.txt
line 104
ZNF618 5808-mir-125b.txt
line 408
ZNF618 5809-mir-128.txt
line 250
ZNF618 5810-mir-143.txt
line 159
ZNF618 5811-mir-144.txt
line 376
ZNF618 5812-mir-155.txt
line 106

NEO1 5802-mir-9.txt
line 806
NEO1 5808-mir-125b.txt
line 277
NEO1 5809-mir-128.txt

line 211

RAP1B 5802-mir-9.txt
line 809
RAP1B 5809-mir-128.txt
line 220
RAP1B 5812-mir-155.txt
line 237

hCG_1757335 5802-mir-9.txt
line 810
hCG_1757335 5809-mir-128.txt
line 173
hCG_1757335 5812-mir-155.txt
line 230

FOXO1 5802-mir-9.txt
line 815
FOXO1 5809-mir-128.txt
line 198
FOXO1 5811-mir-144.txt
line 447

SERBP1 5802-mir-9.txt
line 816
SERBP1 5803-mir-16.txt
line 151
SERBP1 5809-mir-128.txt
line 731

tcag7.1228 5802-mir-9.txt
line 818
tcag7.1228 5803-mir-16.txt
line 6
tcag7.1228 5804-miR-21.txt
line 32
tcag7.1228 5809-mir-128.txt
line 266
tcag7.1228 5811-mir-144.txt
line 21

LIN7C 5802-mir-9.txt
line 819
LIN7C 5805-mir-22.txt

line 27
LIN7C 5809-mir-128.txt
line 430

SFRS12IP1 5802-mir-9.txt
line 821
SFRS12IP1 5808-mir-125b.txt
line 285

DCTN1 5802-mir-9.txt
line 822
DCTN1 5808-mir-125b.txt
line 363

KSR1 5802-mir-9.txt
line 823
KSR1 5812-mir-155.txt
line 160

PHF13 5802-mir-9.txt
line 824
PHF13 5805-mir-22.txt
line 259

CBX7 5802-mir-9.txt
line 825
CBX7 5808-mir-125b.txt
line 307

FBXO33 5802-mir-9.txt
line 827
FBXO33 5809-mir-128.txt
line 133
FBXO33 5812-mir-155.txt
line 67

UBR1 5802-mir-9.txt
line 828
UBR1 5809-mir-128.txt
line 84

FOXO3 5802-mir-9.txt
line 829
FOXO3 5806-mir-29b.txt

line 92

PTGFRN 5802-mir-9.txt
line 830
PTGFRN 5809-mir-128.txt
line 494
PTGFRN 5811-mir-144.txt
line 348

DDX3X 5802-mir-9.txt
line 831
DDX3X 5803-mir-16.txt
line 80

NFASC 5802-mir-9.txt
line 832
NFASC 5812-mir-155.txt
line 242

PHF15 5802-mir-9.txt
line 833
PHF15 5808-mir-125b.txt
line 98
PHF15 5809-mir-128.txt
line 773

PSCD3 5802-mir-9.txt
line 834
PSCD3 5805-mir-22.txt
line 30
PSCD3 5811-mir-144.txt
line 250

ZNF236 5802-mir-9.txt
line 836
ZNF236 5812-mir-155.txt
line 62

JHDM1D 5802-mir-9.txt
line 837
JHDM1D 5809-mir-128.txt
line 32
JHDM1D 5811-mir-144.txt
line 158

JHDM1D 5812-mir-155.txt
line 79

RIMS3 5802-mir-9.txt
line 838
RIMS3 5803-mir-16.txt
line 231

RCAN2 5802-mir-9.txt
line 846
RCAN2 5809-mir-128.txt
line 77

MYO10 5802-mir-9.txt
line 847
MYO10 5812-mir-155.txt
line 133

NFIB 5802-mir-9.txt
line 848
NFIB 5804-miR-21.txt
line 24
NFIB 5805-mir-22.txt
line 304
NFIB 5806-mir-29b.txt
line 119
NFIB 5809-mir-128.txt
line 729

CBX6 5802-mir-9.txt
line 850
CBX6 5805-mir-22.txt
line 247
CBX6 5806-mir-29b.txt
line 93

RAB5B 5802-mir-9.txt
line 851
RAB5B 5805-mir-22.txt
line 60

DCP1A 5802-mir-9.txt
line 852
DCP1A 5808-mir-125b.txt

line 371
DCP1A 5809-mir-128.txt
line 124

CSNK1G2 5802-mir-9.txt
line 858
CSNK1G2 5812-mir-155.txt
line 125

TSPAN9 5802-mir-9.txt
line 860
TSPAN9 5803-mir-16.txt
line 287

IGF2R 5802-mir-9.txt
line 861
IGF2R 5811-mir-144.txt
line 359

SRF 5802-mir-9.txt
line 862
SRF 5805-mir-22.txt
line 323
SRF 5808-mir-125b.txt
line 357
SRF 5811-mir-144.txt
line 631

ZFHX3 5802-mir-9.txt
line 863
ZFHX3 5809-mir-128.txt
line 226
ZFHX3 5811-mir-144.txt
line 567

ACVR1B 5802-mir-9.txt
line 864
ACVR1B 5805-mir-22.txt
line 282

MTPN 5802-mir-9.txt
line 868
MTPN 5811-mir-144.txt
line 340

OTUD4 5802-mir-9.txt
line 869
OTUD4 5803-mir-16.txt
line 183
OTUD4 5810-mir-143.txt
line 86

KIAA0495 5802-mir-9.txt
line 870
KIAA0495 5812-mir-155.txt
line 49

PHF21A 5802-mir-9.txt
line 872
PHF21A 5803-mir-16.txt
line 290

SMPD3 5802-mir-9.txt
line 875
SMPD3 5809-mir-128.txt
line 521
SMPD3 5811-mir-144.txt
line 293

PGAP1 5802-mir-9.txt
line 879
PGAP1 5809-mir-128.txt
line 308

MYO1D 5802-mir-9.txt
line 880
MYO1D 5812-mir-155.txt
line 64

C11orf41 5802-mir-9.txt
line 881
C11orf41 5806-mir-29b.txt
line 101
C11orf41 5809-mir-128.txt
line 279
C11orf41 5811-mir-144.txt
line 104

DOT1L 5802-mir-9.txt
line 883
DOT1L 5809-mir-128.txt
line 235

ZNF362 5802-mir-9.txt
line 884
ZNF362 5811-mir-144.txt
line 474

SH3PXD2A 5802-mir-9.txt
line 885
SH3PXD2A 5805-mir-22.txt
line 287
SH3PXD2A 5806-mir-29b.txt
line 19
SH3PXD2A 5810-mir-143.txt
line 254
SH3PXD2A 5811-mir-144.txt
line 646
SH3PXD2A 5812-mir-155.txt
line 272

SELI 5802-mir-9.txt
line 886
SELI 5810-mir-143.txt
line 75
SELI 5811-mir-144.txt
line 417

HRBL 5802-mir-9.txt
line 888
HRBL 5809-mir-128.txt
line 783

BTG2 5802-mir-9.txt
line 893
BTG2 5803-mir-16.txt
line 177
BTG2 5809-mir-128.txt
line 476

TSC1 5802-mir-9.txt
line 894

TSC1 5809-mir-128.txt
line 362

XYLT1 5802-mir-9.txt
line 895
XYLT1 5811-mir-144.txt
line 22

SYT14 5802-mir-9.txt
line 901
SYT14 5811-mir-144.txt
line 613

SRC 5802-mir-9.txt
line 902
SRC 5811-mir-144.txt
line 381

HDAC4 5802-mir-9.txt
line 903
HDAC4 5805-mir-22.txt
line 300
HDAC4 5806-mir-29b.txt
line 58
HDAC4 5809-mir-128.txt
line 647

KLF13 5802-mir-9.txt
line 904
KLF13 5810-mir-143.txt
line 261

BTBD14B 5802-mir-9.txt
line 907
BTBD14B 5811-mir-144.txt
line 516

MEX3A 5802-mir-9.txt
line 909
MEX3A 5805-mir-22.txt
line 273

RNF44 5802-mir-9.txt
line 911

RNF44 5805-mir-22.txt
line 188
RNF44 5811-mir-144.txt
line 618

BRPF3 5802-mir-9.txt
line 912
BRPF3 5811-mir-144.txt
line 358

GTPBP1 5802-mir-9.txt
line 914
GTPBP1 5803-mir-16.txt
line 235

TNRC6B 5802-mir-9.txt
line 915
TNRC6B 5803-mir-16.txt
line 4
TNRC6B 5805-mir-22.txt
line 182
TNRC6B 5806-mir-29b.txt
line 158
TNRC6B 5809-mir-128.txt
line 174
TNRC6B 5811-mir-144.txt
line 450

ARFIP2 5802-mir-9.txt
line 917
ARFIP2 5805-mir-22.txt
line 115

SNX30 5802-mir-9.txt
line 919
SNX30 5805-mir-22.txt
line 271

EPHB2 5802-mir-9.txt
line 920
EPHB2 5809-mir-128.txt
line 223

LASP1 5802-mir-9.txt

line 921
LASP1 5809-mir-128.txt
line 387
LASP1 5810-mir-143.txt
line 247

IGF2BP1 5802-mir-9.txt
line 923
IGF2BP1 5805-mir-22.txt
line 257

EGR3 5802-mir-9.txt
line 926
EGR3 5804-miR-21.txt
line 83
EGR3 5809-mir-128.txt
line 141

MMP15 5802-mir-9.txt
line 927
MMP15 5811-mir-144.txt
line 641

ZHX3 5802-mir-9.txt
line 928
ZHX3 5803-mir-16.txt
line 99
ZHX3 5806-mir-29b.txt
line 40
ZHX3 5810-mir-143.txt
line 163

PGRMC2 5802-mir-9.txt
line 929
PGRMC2 5811-mir-144.txt
line 96

STXBP1 5802-mir-9.txt
line 930
STXBP1 5810-mir-143.txt
line 105

VPS4B 5802-mir-9.txt
line 932

VPS4B	5808-mir-125b.txt
line 11	
VPS4B	5809-mir-128.txt
line 355	
VPS4B	5811-mir-144.txt
line 276	
ERC2	5802-mir-9.txt
line 935	
ERC2	5809-mir-128.txt
line 187	
MGAT4A	5803-mir-16.txt
line 5	
MGAT4A	5806-mir-29b.txt
line 50	
MGAT4A	5808-mir-125b.txt
line 143	
MGAT4A	5811-mir-144.txt
line 133	
HELZ	5803-mir-16.txt
line 18	
HELZ	5811-mir-144.txt
line 555	
ACVR2B	5803-mir-16.txt
line 19	
ACVR2B	5811-mir-144.txt
line 295	
ACVR2B	5812-mir-155.txt
line 264	
RSBN1	5803-mir-16.txt
line 26	
RSBN1	5805-mir-22.txt
line 21	
RSBN1	5809-mir-128.txt
line 412	
ASH1L	5803-mir-16.txt
line 27	
ASH1L	5810-mir-143.txt
line 169	

ASH1L 5811-mir-144.txt
line 362

SLC12A2 5803-mir-16.txt
line 28
SLC12A2 5811-mir-144.txt
line 3

NUP50 5803-mir-16.txt
line 36
NUP50 5808-mir-125b.txt
line 398

GABARAPL1 5803-mir-16.txt
line 40
GABARAPL1 5810-mir-143.txt
line 120

FAM73A 5803-mir-16.txt
line 41
FAM73A 5808-mir-125b.txt
line 302
FAM73A 5811-mir-144.txt
line 464

MOBKL3 5803-mir-16.txt
line 46
MOBKL3 5811-mir-144.txt
line 35

CD2AP 5803-mir-16.txt
line 47
CD2AP 5809-mir-128.txt
line 649

MAFK 5803-mir-16.txt
line 51
MAFK 5811-mir-144.txt
line 354

FLJ40142 5803-mir-16.txt
line 53
FLJ40142 5811-mir-144.txt
line 285

ZMYM2 5803-mir-16.txt
line 58
ZMYM2 5811-mir-144.txt
line 505
ZMYM2 5812-mir-155.txt
line 206

SMAD5 5803-mir-16.txt
line 62
SMAD5 5809-mir-128.txt
line 416
SMAD5 5811-mir-144.txt
line 426

MYLK 5803-mir-16.txt
line 64
MYLK 5812-mir-155.txt
line 53

FBXW7 5803-mir-16.txt
line 73
FBXW7 5805-mir-22.txt
line 203
FBXW7 5809-mir-128.txt
line 287
FBXW7 5811-mir-144.txt
line 638

SOBP 5803-mir-16.txt
line 81
SOBP 5810-mir-143.txt
line 81
SOBP 5811-mir-144.txt
line 339

TMEM189-UBE2V1 5803-mir-16.txt
line 83
TMEM189-UBE2V1 5809-mir-128.txt
line 671
TMEM189-UBE2V1 5811-mir-144.txt
line 187

UBE2V1 5803-mir-16.txt

line 84
UBE2V1 5809-mir-128.txt
line 672
UBE2V1 5811-mir-144.txt
line 188

NAPEPLD 5803-mir-16.txt
line 87
NAPEPLD 5809-mir-128.txt
line 720

FAT3 5803-mir-16.txt
line 88
FAT3 5811-mir-144.txt
line 12

ABI2 5803-mir-16.txt
line 91
ABI2 5811-mir-144.txt
line 174

CCNT2 5803-mir-16.txt
line 92
CCNT2 5811-mir-144.txt
line 8

TACC1 5803-mir-16.txt
line 93
TACC1 5809-mir-128.txt
line 326

CYP26B1 5803-mir-16.txt
line 100
CYP26B1 5807-mir-99a.txt
line 23
CYP26B1 5812-mir-155.txt
line 213

NARG1 5803-mir-16.txt
line 101
NARG1 5809-mir-128.txt
line 334

HOXA10 5803-mir-16.txt

line 104
HOXA10 5809-mir-128.txt
line 522
HOXA10 5811-mir-144.txt
line 389

SMAD3 5803-mir-16.txt
line 111
SMAD3 5810-mir-143.txt
line 231

YOD1 5803-mir-16.txt
line 112
YOD1 5808-mir-125b.txt
line 328

ZCCHC3 5803-mir-16.txt
line 118
ZCCHC3 5804-miR-21.txt
line 84

BDNF 5803-mir-16.txt
line 121
BDNF 5812-mir-155.txt
line 191

B4GALT1 5803-mir-16.txt
line 123
B4GALT1 5808-mir-125b.txt
line 351

WDR82 5803-mir-16.txt
line 130
WDR82 5805-mir-22.txt
line 78

CCND1 5803-mir-16.txt
line 132
CCND1 5812-mir-155.txt
line 261

SVEP1 5803-mir-16.txt
line 133
SVEP1 5810-mir-143.txt

line 193

ATP13A3 5803-mir-16.txt
line 135
ATP13A3 5808-mir-125b.txt
line 274

LRP1B 5803-mir-16.txt
line 138
LRP1B 5812-mir-155.txt
line 21

C1orf9 5803-mir-16.txt
line 139
C1orf9 5809-mir-128.txt
line 484

RCOR3 5803-mir-16.txt
line 141
RCOR3 5809-mir-128.txt
line 7

BAG5 5803-mir-16.txt
line 146
BAG5 5812-mir-155.txt
line 37

ZNF654 5803-mir-16.txt
line 148
ZNF654 5811-mir-144.txt
line 70

ZCCHC2 5803-mir-16.txt
line 149
ZCCHC2 5811-mir-144.txt
line 155

IRF4 5803-mir-16.txt
line 152
IRF4 5809-mir-128.txt
line 10

GRAMD3 5803-mir-16.txt
line 153

GRAMD3 5804-miR-21.txt
line 26

RICTOR 5803-mir-16.txt
line 154
RICTOR 5809-mir-128.txt
line 739
RICTOR 5810-mir-143.txt
line 108
RICTOR 5812-mir-155.txt
line 23

JARID2 5803-mir-16.txt
line 158
JARID2 5805-mir-22.txt
line 290
JARID2 5812-mir-155.txt
line 4

EPB41L4B 5803-mir-16.txt
line 159
EPB41L4B 5805-mir-22.txt
line 166

AMOT 5803-mir-16.txt
line 164
AMOT 5805-mir-22.txt
line 208

PARD6B 5803-mir-16.txt
line 165
PARD6B 5809-mir-128.txt
line 520

GALNT1 5803-mir-16.txt
line 169
GALNT1 5811-mir-144.txt
line 407

C20orf39 5803-mir-16.txt
line 171
C20orf39 5809-mir-128.txt
line 547

CBFA2T3 5803-mir-16.txt
line 173
CBFA2T3 5809-mir-128.txt
line 696

ACSL4 5803-mir-16.txt
line 178
ACSL4 5811-mir-144.txt
line 265

YWHAQ 5803-mir-16.txt
line 179
YWHAQ 5813-mir-200c.txt
line 23

ARRDC4 5803-mir-16.txt
line 180
ARRDC4 5806-mir-29b.txt
line 153
ARRDC4 5809-mir-128.txt
line 27

PHC3 5803-mir-16.txt
line 181
PHC3 5812-mir-155.txt
line 14

EVI5 5803-mir-16.txt
line 185
EVI5 5809-mir-128.txt
line 568

COPS2 5803-mir-16.txt
line 191
COPS2 5811-mir-144.txt
line 522

FRYL 5803-mir-16.txt
line 192
FRYL 5809-mir-128.txt
line 121

LPHN2 5803-mir-16.txt
line 193

```
LPHN2                     5811-mir-144.txt
line   590

NAV1                      5803-mir-16.txt
line   194
NAV1                      5806-mir-29b.txt
line    29

KIAA1333                  5803-mir-16.txt
line   195
KIAA1333                  5812-mir-155.txt
line    10

ZNF367                    5803-mir-16.txt
line   204
ZNF367                    5804-miR-21.txt
line    53

KPNA3                     5803-mir-16.txt
line   216
KPNA3                     5811-mir-144.txt
line   518

PHLPPL                    5803-mir-16.txt
line   217
PHLPPL                    5809-mir-128.txt
line   307

FREQ                      5803-mir-16.txt
line   227
FREQ                      5809-mir-128.txt
line   276
FREQ                      5811-mir-144.txt
line   550

PRPF38A                   5803-mir-16.txt
line   228
PRPF38A                   5805-mir-22.txt
line    35

CHD7                      5803-mir-16.txt
line   229
CHD7                      5805-mir-22.txt
line   190
```

CHD7		5812-mir-155.txt
line	246	
PELI2		5803-mir-16.txt
line	230	
PELI2		5808-mir-125b.txt
line	289	
PELI2		5809-mir-128.txt
line	548	
SRP72		5803-mir-16.txt
line	232	
SRP72		5809-mir-128.txt
line	268	
CTDSPL		5803-mir-16.txt
line	234	
CTDSPL		5809-mir-128.txt
line	769	
FRS2		5803-mir-16.txt
line	238	
FRS2		5810-mir-143.txt
line	82	
FRS2		5811-mir-144.txt
line	54	
NUP210		5803-mir-16.txt
line	239	
NUP210		5808-mir-125b.txt
line	15	
NUP210		5809-mir-128.txt
line	552	
BACH2		5803-mir-16.txt
line	241	
BACH2		5811-mir-144.txt
line	47	
PLEKHH1		5803-mir-16.txt
line	242	
PLEKHH1		5809-mir-128.txt
line	734	

HPCAL4 5803-mir-16.txt
line 244
HPCAL4 5806-mir-29b.txt
line 41

PIM1 5803-mir-16.txt
line 245
PIM1 5811-mir-144.txt
line 214

CDV3 5803-mir-16.txt
line 247
CDV3 5811-mir-144.txt
line 149

ESRRG 5803-mir-16.txt
line 248
ESRRG 5810-mir-143.txt
line 222
ESRRG 5811-mir-144.txt
line 332

MMD 5803-mir-16.txt
line 249
MMD 5809-mir-128.txt
line 413

ABTB2 5803-mir-16.txt
line 251
ABTB2 5811-mir-144.txt
line 461

PURB 5803-mir-16.txt
line 252
PURB 5804-miR-21.txt
line 76
PURB 5805-mir-22.txt
line 142
PURB 5811-mir-144.txt
line 473

KIF3B 5803-mir-16.txt
line 256
KIF3B 5810-mir-143.txt

line 47

DLL4 5803-mir-16.txt
line 257
DLL4 5809-mir-128.txt
line 602

FOXK1 5803-mir-16.txt
line 260
FOXK1 5812-mir-155.txt
line 280

ADAMTS18 5803-mir-16.txt
line 261
ADAMTS18 5806-mir-29b.txt
line 53

MAP3K7 5803-mir-16.txt
line 264
MAP3K7 5810-mir-143.txt
line 62

MED1 5803-mir-16.txt
line 267
MED1 5809-mir-128.txt
line 749

CAB39 5803-mir-16.txt
line 277
CAB39 5812-mir-155.txt
line 154

ZC3H12B 5803-mir-16.txt
line 278
ZC3H12B 5809-mir-128.txt
line 747

TET3 5803-mir-16.txt
line 286
TET3 5808-mir-125b.txt
line 333
TET3 5811-mir-144.txt
line 628

GATAD2B 5804-miR-21.txt
line 1
GATAD2B 5812-mir-155.txt
line 273

SCML2 5804-miR-21.txt
line 3
SCML2 5806-mir-29b.txt
line 24

SATB1 5804-miR-21.txt
line 4
SATB1 5812-mir-155.txt
line 168

TIAM1 5804-miR-21.txt
line 5
TIAM1 5805-mir-22.txt
line 206
TIAM1 5806-mir-29b.txt
line 107

THRB 5804-miR-21.txt
line 10
THRB 5809-mir-128.txt
line 585

FGF1 5804-miR-21.txt
line 20
FGF1 5810-mir-143.txt
line 123

TIMP3 5804-miR-21.txt
line 23
TIMP3 5811-mir-144.txt
line 606

SRL 5804-miR-21.txt
line 27
SRL 5809-mir-128.txt
line 612

JAG1 5804-miR-21.txt
line 29

JAG1 5809-mir-128.txt
line 275

LRP6 5804-miR-21.txt
line 31
LRP6 5806-mir-29b.txt
line 16
LRP6 5809-mir-128.txt
line 530

MEIS1 5804-miR-21.txt
line 36
MEIS1 5811-mir-144.txt
line 345
MEIS1 5812-mir-155.txt
line 128

TET1 5804-miR-21.txt
line 43
TET1 5809-mir-128.txt
line 299
TET1 5810-mir-143.txt
line 56

ASPN 5804-miR-21.txt
line 45
ASPN 5811-mir-144.txt
line 277

SPRY2 5804-miR-21.txt
line 46
SPRY2 5809-mir-128.txt
line 593

PAG1 5804-miR-21.txt
line 47
PAG1 5806-mir-29b.txt
line 46
PAG1 5811-mir-144.txt
line 231

EHD1 5804-miR-21.txt
line 49
EHD1 5812-mir-155.txt

line 249

PIK3R1 5804-miR-21.txt
line 50
PIK3R1 5809-mir-128.txt
line 271
PIK3R1 5812-mir-155.txt
line 211

ARHGEF12 5804-miR-21.txt
line 56
ARHGEF12 5805-mir-22.txt
line 94

SOCS6 5804-miR-21.txt
line 58
SOCS6 5809-mir-128.txt
line 60
SOCS6 5811-mir-144.txt
line 291
SOCS6 5812-mir-155.txt
line 225

SNTB2 5804-miR-21.txt
line 61
SNTB2 5811-mir-144.txt
line 76

BOLL 5804-miR-21.txt
line 62
BOLL 5805-mir-22.txt
line 328

ATPAF1 5804-miR-21.txt
line 63
ATPAF1 5809-mir-128.txt
line 698

CBX4 5804-miR-21.txt
line 64
CBX4 5811-mir-144.txt
line 228

FBXO11 5804-miR-21.txt

line 69
FBXO11 5812-mir-155.txt
line 149

GLT8D3 5804-miR-21.txt
line 72
GLT8D3 5809-mir-128.txt
line 40
GLT8D3 5810-mir-143.txt
line 8

ACVR2A 5804-miR-21.txt
line 78
ACVR2A 5809-mir-128.txt
line 88

SOX7 5804-miR-21.txt
line 79
SOX7 5809-mir-128.txt
line 181

STK40 5804-miR-21.txt
line 81
STK40 5809-mir-128.txt
line 379

COL4A1 5804-miR-21.txt
line 82
COL4A1 5806-mir-29b.txt
line 10

PI15 5804-miR-21.txt
line 90
PI15 5806-mir-29b.txt
line 1
PI15 5807-mir-99a.txt
line 20

PRKCE 5804-miR-21.txt
line 93
PRKCE 5810-mir-143.txt
line 127
PRKCE 5811-mir-144.txt
line 463

ZBTB47 5804-miR-21.txt
line 99
ZBTB47 5806-mir-29b.txt
line 79
ZBTB47 5808-mir-125b.txt
line 393

DCUN1D3 5804-miR-21.txt
line 101
DCUN1D3 5812-mir-155.txt
line 247

DNAJC16 5804-miR-21.txt
line 102
DNAJC16 5805-mir-22.txt
line 154

ZADH2 5804-miR-21.txt
line 104
ZADH2 5809-mir-128.txt
line 656

FUT9 5805-mir-22.txt
line 1
FUT9 5809-mir-128.txt
line 390
FUT9 5812-mir-155.txt
line 80

TET2 5805-mir-22.txt
line 4
TET2 5809-mir-128.txt
line 165

H3F3B 5805-mir-22.txt
line 5
H3F3B 5809-mir-128.txt
line 28

LAMC1 5805-mir-22.txt
line 9
LAMC1 5806-mir-29b.txt
line 89

GIGYF2 5805-mir-22.txt
line 11
GIGYF2 5809-mir-128.txt
line 113
GIGYF2 5810-mir-143.txt
line 32

LOC440093 5805-mir-22.txt
line 14
LOC440093 5809-mir-128.txt
line 91

CLIC4 5805-mir-22.txt
line 16
CLIC4 5809-mir-128.txt
line 225

TP53INP1 5805-mir-22.txt
line 19
TP53INP1 5808-mir-125b.txt
line 116
TP53INP1 5811-mir-144.txt
line 527
TP53INP1 5812-mir-155.txt
line 85

VEZF1 5805-mir-22.txt
line 22
VEZF1 5813-mir-200c.txt
line 31

ERBB3 5805-mir-22.txt
line 36
ERBB3 5810-mir-143.txt
line 113

FAM125B 5805-mir-22.txt
line 44
FAM125B 5809-mir-128.txt
line 244

PTEN 5805-mir-22.txt
line 47

PTEN 5811-mir-144.txt
line 367

CSF1R 5805-mir-22.txt
line 51
CSF1R 5812-mir-155.txt
line 136

CHD9 5805-mir-22.txt
line 52
CHD9 5812-mir-155.txt
line 68

KIAA1486 5805-mir-22.txt
line 53
KIAA1486 5811-mir-144.txt
line 38

MAGI2 5805-mir-22.txt
line 54
MAGI2 5811-mir-144.txt
line 243

CAV3 5805-mir-22.txt
line 69
CAV3 5811-mir-144.txt
line 565

C1orf21 5805-mir-22.txt
line 72
C1orf21 5809-mir-128.txt
line 315

BCL9L 5805-mir-22.txt
line 73
BCL9L 5806-mir-29b.txt
line 154
BCL9L 5811-mir-144.txt
line 612

PHACTR2 5805-mir-22.txt
line 77
PHACTR2 5811-mir-144.txt
line 380

PAIP2B 5805-mir-22.txt
line 81
PAIP2B 5809-mir-128.txt
line 679

PPP1R9A 5805-mir-22.txt
line 84
PPP1R9A 5809-mir-128.txt
line 636

YWHAZ 5805-mir-22.txt
line 85
YWHAZ 5812-mir-155.txt
line 148

COPS7B 5805-mir-22.txt
line 86
COPS7B 5808-mir-125b.txt
line 301

SNAI1 5805-mir-22.txt
line 92
SNAI1 5809-mir-128.txt
line 711

RFXANK 5805-mir-22.txt
line 99
RFXANK 5808-mir-125b.txt
line 68

SATB2 5805-mir-22.txt
line 100
SATB2 5809-mir-128.txt
line 514

LRRC1 5805-mir-22.txt
line 101
LRRC1 5811-mir-144.txt
line 398

MON2 5805-mir-22.txt
line 110
MON2 5811-mir-144.txt

line 553

MAPK14 5805-mir-22.txt
line 112
MAPK14 5809-mir-128.txt
line 193

STK39 5805-mir-22.txt
line 114
STK39 5809-mir-128.txt
line 471

TRIB2 5805-mir-22.txt
line 118
TRIB2 5808-mir-125b.txt
line 239

FBXO46 5805-mir-22.txt
line 125
FBXO46 5810-mir-143.txt
line 221

EDA 5805-mir-22.txt
line 127
EDA 5811-mir-144.txt
line 275

BAT2D1 5805-mir-22.txt
line 131
BAT2D1 5806-mir-29b.txt
line 27
BAT2D1 5811-mir-144.txt
line 569

C14orf83 5805-mir-22.txt
line 141
C14orf83 5806-mir-29b.txt
line 157
C14orf83 5808-mir-125b.txt
line 403

SV2A 5805-mir-22.txt
line 146
SV2A 5809-mir-128.txt

```
line   251

SLC6A1                    5805-mir-22.txt
line   147
SLC6A1                    5809-mir-128.txt
line    31

MEIS2                     5805-mir-22.txt
line   153
MEIS2                     5809-mir-128.txt
line   610
MEIS2                     5811-mir-144.txt
line    94

NUDT4                     5805-mir-22.txt
line   158
NUDT4                     5812-mir-155.txt
line   215

ZCCHC14                   5805-mir-22.txt
line   159
ZCCHC14                   5811-mir-144.txt
line   510

NECAP1                    5805-mir-22.txt
line   162
NECAP1                    5810-mir-143.txt
line    53

MECP2                     5805-mir-22.txt
line   164
MECP2                     5812-mir-155.txt
line   274

ZMYM4                     5805-mir-22.txt
line   165
ZMYM4                     5809-mir-128.txt
line   560

KSR2                      5805-mir-22.txt
line   167
KSR2                      5808-mir-125b.txt
line   225
KSR2                      5809-mir-128.txt
```

line 188

MAP2K4 5805-mir-22.txt
line 171
MAP2K4 5809-mir-128.txt
line 56

PTPN9 5805-mir-22.txt
line 176
PTPN9 5809-mir-128.txt
line 377
PTPN9 5811-mir-144.txt
line 122

EDC3 5805-mir-22.txt
line 179
EDC3 5811-mir-144.txt
line 542

NXT2 5805-mir-22.txt
line 180
NXT2 5809-mir-128.txt
line 134

PRDM16 5805-mir-22.txt
line 181
PRDM16 5809-mir-128.txt
line 353
PRDM16 5811-mir-144.txt
line 185

SPTY2D1 5805-mir-22.txt
line 183
SPTY2D1 5809-mir-128.txt
line 85
SPTY2D1 5812-mir-155.txt
line 198

TGFBR1 5805-mir-22.txt
line 199
TGFBR1 5809-mir-128.txt
line 351

EPC1 5805-mir-22.txt

line 202	
EPC1	5806-mir-29b.txt
line 115	
ADCY6	5805-mir-22.txt
line 204	
ADCY6	5809-mir-128.txt
line 598	
PPP1R16B	5805-mir-22.txt
line 209	
PPP1R16B	5811-mir-144.txt
line 219	
TRIM67	5805-mir-22.txt
line 212	
TRIM67	5809-mir-128.txt
line 163	
KIAA1274	5805-mir-22.txt
line 213	
KIAA1274	5812-mir-155.txt
line 48	
PLAGL2	5805-mir-22.txt
line 215	
PLAGL2	5808-mir-125b.txt
line 395	
PLAGL2	5809-mir-128.txt
line 313	
PLAGL2	5810-mir-143.txt
line 242	
JMJD3	5805-mir-22.txt
line 216	
JMJD3	5811-mir-144.txt
line 637	
CUL3	5805-mir-22.txt
line 219	
CUL3	5811-mir-144.txt
line 283	
HNRNPA3	5805-mir-22.txt

line 224
HNRNPA3 5812-mir-155.txt
line 36

GPR107 5805-mir-22.txt
line 226
GPR107 5808-mir-125b.txt
line 120

SLC6A17 5805-mir-22.txt
line 227
SLC6A17 5808-mir-125b.txt
line 195
SLC6A17 5811-mir-144.txt
line 615

SCAMP1 5805-mir-22.txt
line 231
SCAMP1 5811-mir-144.txt
line 509

UBE2NL 5805-mir-22.txt
line 235
UBE2NL 5809-mir-128.txt
line 186

SAMD12 5805-mir-22.txt
line 237
SAMD12 5809-mir-128.txt
line 407
SAMD12 5811-mir-144.txt
line 267
SAMD12 5812-mir-155.txt
line 123

TTC9 5805-mir-22.txt
line 242
TTC9 5809-mir-128.txt
line 317

ITGB8 5805-mir-22.txt
line 245
ITGB8 5810-mir-143.txt
line 69

ITGB8 5811-mir-144.txt
line 496

DDX6 5805-mir-22.txt
line 251
DDX6 5809-mir-128.txt
line 504
DDX6 5810-mir-143.txt
line 232

NPNT 5805-mir-22.txt
line 254
NPNT 5811-mir-144.txt
line 400

NCOA1 5805-mir-22.txt
line 256
NCOA1 5809-mir-128.txt
line 653

UBE2N 5805-mir-22.txt
line 258
UBE2N 5809-mir-128.txt
line 194

WSCD1 5805-mir-22.txt
line 260
WSCD1 5810-mir-143.txt
line 206

ETV1 5805-mir-22.txt
line 261
ETV1 5811-mir-144.txt
line 556
ETV1 5812-mir-155.txt
line 266

ERBB4 5805-mir-22.txt
line 268
ERBB4 5811-mir-144.txt
line 89

NFAT5 5805-mir-22.txt
line 272

NFAT5 5809-mir-128.txt
line 630
NFAT5 5812-mir-155.txt
line 254

WDFY3 5805-mir-22.txt
line 277
WDFY3 5811-mir-144.txt
line 310

USP46 5805-mir-22.txt
line 278
USP46 5809-mir-128.txt
line 622
USP46 5810-mir-143.txt
line 184

BRSK2 5805-mir-22.txt
line 285
BRSK2 5808-mir-125b.txt
line 405
BRSK2 5811-mir-144.txt
line 639

SLC16A2 5805-mir-22.txt
line 286
SLC16A2 5806-mir-29b.txt
line 142
SLC16A2 5810-mir-143.txt
line 65

ZNF609 5805-mir-22.txt
line 293
ZNF609 5806-mir-29b.txt
line 145

SNX18 5805-mir-22.txt
line 295
SNX18 5809-mir-128.txt
line 395

AKAP6 5805-mir-22.txt
line 297
AKAP6 5810-mir-143.txt

line 11

TUB 5805-mir-22.txt
line 301
TUB 5809-mir-128.txt
line 65
TUB 5810-mir-143.txt
line 12

TLK2 5805-mir-22.txt
line 303
TLK2 5808-mir-125b.txt
line 304
TLK2 5809-mir-128.txt
line 470
TLK2 5810-mir-143.txt
line 199

OLA1 5805-mir-22.txt
line 306
OLA1 5811-mir-144.txt
line 377

KCNC1 5805-mir-22.txt
line 310
KCNC1 5810-mir-143.txt
line 248

SP1 5805-mir-22.txt
line 311
SP1 5806-mir-29b.txt
line 134
SP1 5809-mir-128.txt
line 202
SP1 5812-mir-155.txt
line 24

KIAA0556 5805-mir-22.txt
line 315
KIAA0556 5811-mir-144.txt
line 647

OLFML2A 5805-mir-22.txt
line 317

OLFML2A 5808-mir-125b.txt
line 21

ZNF827 5805-mir-22.txt
line 320
ZNF827 5809-mir-128.txt
line 480

VASH1 5805-mir-22.txt
line 325
VASH1 5810-mir-143.txt
line 3

UBE2G1 5805-mir-22.txt
line 329
UBE2G1 5808-mir-125b.txt
line 94
UBE2G1 5811-mir-144.txt
line 210

HBP1 5806-mir-29b.txt
line 3
HBP1 5812-mir-155.txt
line 96

KIAA2022 5806-mir-29b.txt
line 4
KIAA2022 5810-mir-143.txt
line 110

ELN 5806-mir-29b.txt
line 6
ELN 5811-mir-144.txt
line 622

RIT1 5806-mir-29b.txt
line 18
RIT1 5808-mir-125b.txt
line 35

SS18L1 5806-mir-29b.txt
line 23
SS18L1 5809-mir-128.txt
line 618

TXNDC4 5806-mir-29b.txt
line 35
TXNDC4 5809-mir-128.txt
line 398

ADAMTS17 5806-mir-29b.txt
line 37
ADAMTS17 5811-mir-144.txt
line 216

C5orf13 5806-mir-29b.txt
line 38
C5orf13 5809-mir-128.txt
line 129

MAFB 5806-mir-29b.txt
line 42
MAFB 5812-mir-155.txt
line 219

ZFP36L1 5806-mir-29b.txt
line 47
ZFP36L1 5809-mir-128.txt
line 183

JARID1B 5806-mir-29b.txt
line 48
JARID1B 5812-mir-155.txt
line 105

GNA13 5806-mir-29b.txt
line 49
GNA13 5809-mir-128.txt
line 429

BCL11B 5806-mir-29b.txt
line 52
BCL11B 5811-mir-144.txt
line 607

GNG12 5806-mir-29b.txt
line 55
GNG12 5809-mir-128.txt

line 423

CEP68 5806-mir-29b.txt
line 56
CEP68 5811-mir-144.txt
line 206

PAIP2 5806-mir-29b.txt
line 59
PAIP2 5809-mir-128.txt
line 13
PAIP2 5813-mir-200c.txt
line 14

BCORL1 5806-mir-29b.txt
line 70
BCORL1 5809-mir-128.txt
line 168
BCORL1 5810-mir-143.txt
line 183
BCORL1 5812-mir-155.txt
line 229

KIAA0644 5806-mir-29b.txt
line 71
KIAA0644 5808-mir-125b.txt
line 87
KIAA0644 5809-mir-128.txt
line 30

AMMECR1L 5806-mir-29b.txt
line 72
AMMECR1L 5809-mir-128.txt
line 203

RARB 5806-mir-29b.txt
line 75
RARB 5811-mir-144.txt
line 25

C6orf168 5806-mir-29b.txt
line 76
C6orf168 5809-mir-128.txt
line 579

C6orf168 5811-mir-144.txt
line 85

FRAS1 5806-mir-29b.txt
line 77
FRAS1 5808-mir-125b.txt
line 359

MGC21874 5806-mir-29b.txt
line 82
MGC21874 5808-mir-125b.txt
line 385
MGC21874 5812-mir-155.txt
line 171

LCORL 5806-mir-29b.txt
line 83
LCORL 5812-mir-155.txt
line 28

LL22NC03-75B3.6 5806-mir-29b.txt
line 87
LL22NC03-75B3.6 5808-mir-125b.txt
line 163

RNF165 5806-mir-29b.txt
line 90
RNF165 5810-mir-143.txt
line 227

REPS2 5806-mir-29b.txt
line 97
REPS2 5812-mir-155.txt
line 151

C13orf23 5806-mir-29b.txt
line 98
C13orf23 5811-mir-144.txt
line 465

YWHAE 5806-mir-29b.txt
line 102
YWHAE 5812-mir-155.txt
line 258

CNR1 5806-mir-29b.txt
line 111
CNR1 5809-mir-128.txt
line 369

KIAA1602 5806-mir-29b.txt
line 116
KIAA1602 5808-mir-125b.txt
line 183

C18orf62 5806-mir-29b.txt
line 117
C18orf62 5811-mir-144.txt
line 551

GPR85 5806-mir-29b.txt
line 125
GPR85 5811-mir-144.txt
line 545

HNRNPF 5806-mir-29b.txt
line 126
HNRNPF 5809-mir-128.txt
line 631
HNRNPF 5811-mir-144.txt
line 248

EPHA10 5806-mir-29b.txt
line 133
EPHA10 5808-mir-125b.txt
line 378

KCNJ12 5806-mir-29b.txt
line 138
KCNJ12 5808-mir-125b.txt
line 245

FAM53C 5806-mir-29b.txt
line 151
FAM53C 5808-mir-125b.txt
line 327

RAB6B 5806-mir-29b.txt

line 155
RAB6B 5808-mir-125b.txt
line 154

CCDC28B 5806-mir-29b.txt
line 156
CCDC28B 5809-mir-128.txt
line 664

KBTBD8 5807-mir-99a.txt
line 2
KBTBD8 5811-mir-144.txt
line 272

TTC39A 5807-mir-99a.txt
line 4
TTC39A 5809-mir-128.txt
line 94

ADCY1 5807-mir-99a.txt
line 7
ADCY1 5808-mir-125b.txt
line 262
ADCY1 5811-mir-144.txt
line 529

GRHL1 5807-mir-99a.txt
line 15
GRHL1 5808-mir-125b.txt
line 273

ZBTB7A 5807-mir-99a.txt
line 24
ZBTB7A 5808-mir-125b.txt
line 263

NAIF1 5808-mir-125b.txt
line 4
NAIF1 5809-mir-128.txt
line 685

BAP1 5808-mir-125b.txt
line 17
BAP1 5813-mir-200c.txt

line 7

MEGF9 5808-mir-125b.txt
line 19
MEGF9 5810-mir-143.txt
line 155
MEGF9 5811-mir-144.txt
line 253

PODXL 5808-mir-125b.txt
line 26
PODXL 5811-mir-144.txt
line 324

SOX11 5808-mir-125b.txt
line 42
SOX11 5809-mir-128.txt
line 627
SOX11 5811-mir-144.txt
line 595
SOX11 5812-mir-155.txt
line 121

CYP24A1 5808-mir-125b.txt
line 43
CYP24A1 5811-mir-144.txt
line 120

RBAK 5808-mir-125b.txt
line 49
RBAK 5812-mir-155.txt
line 7

LBH 5808-mir-125b.txt
line 60
LBH 5809-mir-128.txt
line 71
LBH 5810-mir-143.txt
line 179

NXF1 5808-mir-125b.txt
line 67
NXF1 5809-mir-128.txt
line 763

TDG 5808-mir-125b.txt
line 70
TDG 5811-mir-144.txt
line 554

MAP3K10 5808-mir-125b.txt
line 74
MAP3K10 5812-mir-155.txt
line 152

SLC35A4 5808-mir-125b.txt
line 79
SLC35A4 5810-mir-143.txt
line 173

SLC25A15 5808-mir-125b.txt
line 88
SLC25A15 5810-mir-143.txt
line 76

SYVN1 5808-mir-125b.txt
line 91
SYVN1 5812-mir-155.txt
line 276

SLC4A4 5808-mir-125b.txt
line 101
SLC4A4 5811-mir-144.txt
line 502

DIRAS1 5808-mir-125b.txt
line 110
DIRAS1 5809-mir-128.txt
line 255

KLC2 5808-mir-125b.txt
line 122
KLC2 5810-mir-143.txt
line 152

NT5DC1 5808-mir-125b.txt
line 124
NT5DC1 5811-mir-144.txt

line 29

BMPR1B 5808-mir-125b.txt
line 129
BMPR1B 5811-mir-144.txt
line 147

SMAD2 5808-mir-125b.txt
line 132
SMAD2 5809-mir-128.txt
line 189
SMAD2 5812-mir-155.txt
line 25

BRWD1 5808-mir-125b.txt
line 134
BRWD1 5812-mir-155.txt
line 174

ALDH1A3 5808-mir-125b.txt
line 135
ALDH1A3 5811-mir-144.txt
line 113

KPNA6 5808-mir-125b.txt
line 142
KPNA6 5809-mir-128.txt
line 770
KPNA6 5811-mir-144.txt
line 640

HCN4 5808-mir-125b.txt
line 147
HCN4 5809-mir-128.txt
line 491

GALNT7 5808-mir-125b.txt
line 165
GALNT7 5809-mir-128.txt
line 230
GALNT7 5811-mir-144.txt
line 583

SLC39A13 5808-mir-125b.txt

line 172
SLC39A13 5809-mir-128.txt
line 303

ECE1 5808-mir-125b.txt
line 174
ECE1 5809-mir-128.txt
line 82
ECE1 5811-mir-144.txt
line 579

BCL2 5808-mir-125b.txt
line 176
BCL2 5810-mir-143.txt
line 189

NCAN 5808-mir-125b.txt
line 177
NCAN 5809-mir-128.txt
line 532

PPME1 5808-mir-125b.txt
line 186
PPME1 5809-mir-128.txt
line 222

SEL1L 5808-mir-125b.txt
line 189
SEL1L 5811-mir-144.txt
line 399

SMCR8 5808-mir-125b.txt
line 191
SMCR8 5809-mir-128.txt
line 695

M6PR 5808-mir-125b.txt
line 197
M6PR 5810-mir-143.txt
line 129

ABCC4 5808-mir-125b.txt
line 206
ABCC4 5810-mir-143.txt

line 132

KCNH7 5808-mir-125b.txt
line 207
KCNH7 5811-mir-144.txt
line 418

HOXB3 5808-mir-125b.txt
line 208
HOXB3 5809-mir-128.txt
line 707

AKAP13 5808-mir-125b.txt
line 209
AKAP13 5810-mir-143.txt
line 154

ELOVL6 5808-mir-125b.txt
line 212
ELOVL6 5809-mir-128.txt
line 322

CDR2L 5808-mir-125b.txt
line 219
CDR2L 5809-mir-128.txt
line 556

ZNF148 5808-mir-125b.txt
line 220
ZNF148 5810-mir-143.txt
line 117
ZNF148 5811-mir-144.txt
line 82

ATXN1 5808-mir-125b.txt
line 233
ATXN1 5811-mir-144.txt
line 56

DOCK3 5808-mir-125b.txt
line 236
DOCK3 5811-mir-144.txt
line 478

FAM65B	5808-mir-125b.txt
line 237	
FAM65B	5809-mir-128.txt
line 637	
PAFAH1B1	5808-mir-125b.txt
line 252	
PAFAH1B1	5811-mir-144.txt
line 48	
STMN3	5808-mir-125b.txt
line 256	
STMN3	5810-mir-143.txt
line 238	
CXorf23	5808-mir-125b.txt
line 265	
CXorf23	5811-mir-144.txt
line 177	
ATP2B3	5808-mir-125b.txt
line 279	
ATP2B3	5812-mir-155.txt
line 137	
SLCO2A1	5808-mir-125b.txt
line 297	
SLCO2A1	5810-mir-143.txt
line 187	
TEGT	5808-mir-125b.txt
line 300	
TEGT	5809-mir-128.txt
line 195	
TEGT	5811-mir-144.txt
line 436	
E2F3	5808-mir-125b.txt
line 306	
E2F3	5809-mir-128.txt
line 742	
E2F2	5808-mir-125b.txt
line 310	

E2F2 5812-mir-155.txt
line 117

BRPF1 5808-mir-125b.txt
line 316
BRPF1 5811-mir-144.txt
line 190

YWHAG 5808-mir-125b.txt
line 337
YWHAG 5811-mir-144.txt
line 118

BACH1 5808-mir-125b.txt
line 347
BACH1 5811-mir-144.txt
line 261
BACH1 5812-mir-155.txt
line 3

PHC2 5808-mir-125b.txt
line 353
PHC2 5809-mir-128.txt
line 570
PHC2 5812-mir-155.txt
line 162

EPB41 5808-mir-125b.txt
line 361
EPB41 5809-mir-128.txt
line 623
EPB41 5810-mir-143.txt
line 259
EPB41 5811-mir-144.txt
line 498

TSPAN14 5808-mir-125b.txt
line 365
TSPAN14 5812-mir-155.txt
line 196

VANGL2 5808-mir-125b.txt
line 366
VANGL2 5809-mir-128.txt

line 161

HMGB3 5808-mir-125b.txt
line 376
HMGB3 5809-mir-128.txt
line 239
HMGB3 5812-mir-155.txt
line 236

SLC23A2 5808-mir-125b.txt
line 394
SLC23A2 5811-mir-144.txt
line 318

BBC3 5808-mir-125b.txt
line 402
BBC3 5810-mir-143.txt
line 258
BBC3 5811-mir-144.txt
line 313

CORO1C 5808-mir-125b.txt
line 406
CORO1C 5809-mir-128.txt
line 411

ETNK2 5808-mir-125b.txt
line 412
ETNK2 5812-mir-155.txt
line 238

AFF4 5809-mir-128.txt
line 15
AFF4 5811-mir-144.txt
line 95

SS18 5809-mir-128.txt
line 17
SS18 5811-mir-144.txt
line 26

FGD6 5809-mir-128.txt
line 21
FGD6 5810-mir-143.txt

line 203
FGD6 5811-mir-144.txt
line 7

SASH1 5809-mir-128.txt
line 73
SASH1 5811-mir-144.txt
line 479

SEC24A 5809-mir-128.txt
line 87
SEC24A 5811-mir-144.txt
line 84

BAZ2B 5809-mir-128.txt
line 95
BAZ2B 5811-mir-144.txt
line 215

SLC7A11 5809-mir-128.txt
line 98
SLC7A11 5810-mir-143.txt
line 28
SLC7A11 5811-mir-144.txt
line 14

MSL-1 5809-mir-128.txt
line 104
MSL-1 5810-mir-143.txt
line 201

MIPOL1 5809-mir-128.txt
line 105
MIPOL1 5810-mir-143.txt
line 71

COL21A1 5809-mir-128.txt
line 115
COL21A1 5812-mir-155.txt
line 115

ABBA-1 5809-mir-128.txt
line 130
ABBA-1 5811-mir-144.txt

line 561

WNK1 5809-mir-128.txt
line 131
WNK1 5812-mir-155.txt
line 252

USP42 5809-mir-128.txt
line 142
USP42 5811-mir-144.txt
line 52

FLJ20309 5809-mir-128.txt
line 147
FLJ20309 5810-mir-143.txt
line 167
FLJ20309 5811-mir-144.txt
line 98

PPP1CC 5809-mir-128.txt
line 153
PPP1CC 5811-mir-144.txt
line 172

C20orf194 5809-mir-128.txt
line 159
C20orf194 5811-mir-144.txt
line 455

C10orf26 5809-mir-128.txt
line 191
C10orf26 5812-mir-155.txt
line 97

FAM126B 5809-mir-128.txt
line 196
FAM126B 5811-mir-144.txt
line 202

ST6GALNAC3 5809-mir-128.txt
line 197
ST6GALNAC3 5811-mir-144.txt
line 409

MED13 5809-mir-128.txt
line 201
MED13 5811-mir-144.txt
line 110

NDST1 5809-mir-128.txt
line 224
NDST1 5813-mir-200c.txt
line 34

SPATA2 5809-mir-128.txt
line 240
SPATA2 5811-mir-144.txt
line 620

PDGFRA 5809-mir-128.txt
line 243
PDGFRA 5810-mir-143.txt
line 228

SLC39A11 5809-mir-128.txt
line 273
SLC39A11 5810-mir-143.txt
line 46

FLJ10404 5809-mir-128.txt
line 278
FLJ10404 5811-mir-144.txt
line 401

CPD 5809-mir-128.txt
line 281
CPD 5810-mir-143.txt
line 77

MCF2L 5809-mir-128.txt
line 286
MCF2L 5811-mir-144.txt
line 244

MET 5809-mir-128.txt
line 290
MET 5811-mir-144.txt
line 630

KIAA1033 5809-mir-128.txt
line 309
KIAA1033 5811-mir-144.txt
line 194

FLRT2 5809-mir-128.txt
line 330
FLRT2 5811-mir-144.txt
line 588

TCF4 5809-mir-128.txt
line 331
TCF4 5812-mir-155.txt
line 22

NRK 5809-mir-128.txt
line 333
NRK 5811-mir-144.txt
line 603

TNRC18 5809-mir-128.txt
line 360
TNRC18 5811-mir-144.txt
line 635

CEP135 5809-mir-128.txt
line 361
CEP135 5811-mir-144.txt
line 312

ZNF652 5809-mir-128.txt
line 363
ZNF652 5812-mir-155.txt
line 5

TAL1 5809-mir-128.txt
line 365
TAL1 5811-mir-144.txt
line 573

BHLHB3 5809-mir-128.txt
line 366
BHLHB3 5811-mir-144.txt

line 233

MED14 5809-mir-128.txt
line 367
MED14 5811-mir-144.txt
line 201

FUBP3 5809-mir-128.txt
line 371
FUBP3 5811-mir-144.txt
line 171

MAPK6 5809-mir-128.txt
line 378
MAPK6 5811-mir-144.txt
line 18

SP4 5809-mir-128.txt
line 389
SP4 5811-mir-144.txt
line 60

TGIF2 5809-mir-128.txt
line 396
TGIF2 5811-mir-144.txt
line 393

RAB11FIP1 5809-mir-128.txt
line 403
RAB11FIP1 5810-mir-143.txt
line 126

MYST2 5809-mir-128.txt
line 406
MYST2 5810-mir-143.txt
line 217
MYST2 5811-mir-144.txt
line 632

TROVE2 5809-mir-128.txt
line 408
TROVE2 5811-mir-144.txt
line 319

RPGRIP1L 5809-mir-128.txt
line 414
RPGRIP1L 5811-mir-144.txt
line 374

NR2F2 5809-mir-128.txt
line 421
NR2F2 5811-mir-144.txt
line 148

SFRP1 5809-mir-128.txt
line 431
SFRP1 5811-mir-144.txt
line 360

UST 5809-mir-128.txt
line 438
UST 5812-mir-155.txt
line 250

HAPLN1 5809-mir-128.txt
line 441
HAPLN1 5811-mir-144.txt
line 525

ITSN2 5809-mir-128.txt
line 444
ITSN2 5811-mir-144.txt
line 343

MYH10 5809-mir-128.txt
line 447
MYH10 5811-mir-144.txt
line 342

SLC39A10 5809-mir-128.txt
line 453
SLC39A10 5810-mir-143.txt
line 208
SLC39A10 5811-mir-144.txt
line 532
SLC39A10 5812-mir-155.txt
line 170

KLF3 5809-mir-128.txt
line 460
KLF3 5811-mir-144.txt
line 238
KLF3 5812-mir-155.txt
line 101
KLF3 5813-mir-200c.txt
line 27

CDH11 5809-mir-128.txt
line 461
CDH11 5811-mir-144.txt
line 335

ZHX1 5809-mir-128.txt
line 465
ZHX1 5811-mir-144.txt
line 218

CCNK 5809-mir-128.txt
line 477
CCNK 5811-mir-144.txt
line 427

SEMA6A 5809-mir-128.txt
line 479
SEMA6A 5811-mir-144.txt
line 264

KLF4 5809-mir-128.txt
line 485
KLF4 5813-mir-200c.txt
line 17

NCOA7 5809-mir-128.txt
line 486
NCOA7 5811-mir-144.txt
line 438

NOVA1 5809-mir-128.txt
line 496
NOVA1 5810-mir-143.txt
line 226
NOVA1 5811-mir-144.txt

line 546
NOVA1 5812-mir-155.txt
line 200

ABCC5 5809-mir-128.txt
line 501
ABCC5 5811-mir-144.txt
line 552

DDAH1 5809-mir-128.txt
line 513
DDAH1 5810-mir-143.txt
line 73

GNS 5809-mir-128.txt
line 516
GNS 5810-mir-143.txt
line 101

N4BP1 5809-mir-128.txt
line 523
N4BP1 5812-mir-155.txt
line 233

PAPPA 5809-mir-128.txt
line 526
PAPPA 5811-mir-144.txt
line 352

C12orf34 5809-mir-128.txt
line 534
C12orf34 5811-mir-144.txt
line 642

SPTBN1 5809-mir-128.txt
line 535
SPTBN1 5811-mir-144.txt
line 448

APPBP2 5809-mir-128.txt
line 540
APPBP2 5811-mir-144.txt
line 50

RNF139 5809-mir-128.txt
line 542
RNF139 5811-mir-144.txt
line 513

TAPT1 5809-mir-128.txt
line 545
TAPT1 5812-mir-155.txt
line 35

LIMK1 5809-mir-128.txt
line 553
LIMK1 5810-mir-143.txt
line 216

ARFGEF1 5809-mir-128.txt
line 554
ARFGEF1 5811-mir-144.txt
line 439

AGRN 5809-mir-128.txt
line 559
AGRN 5811-mir-144.txt
line 626

NOL4 5809-mir-128.txt
line 566
NOL4 5811-mir-144.txt
line 309

KIAA1012 5809-mir-128.txt
line 567
KIAA1012 5810-mir-143.txt
line 95
KIAA1012 5811-mir-144.txt
line 191

PANK1 5809-mir-128.txt
line 569
PANK1 5811-mir-144.txt
line 168

ANKS1A 5809-mir-128.txt
line 581

ANKS1A 5811-mir-144.txt
line 481

ELFN2 5809-mir-128.txt
line 582
ELFN2 5811-mir-144.txt
line 602

ATP6V1A 5809-mir-128.txt
line 586
ATP6V1A 5810-mir-143.txt
line 13
ATP6V1A 5811-mir-144.txt
line 508

UPF1 5809-mir-128.txt
line 592
UPF1 5810-mir-143.txt
line 177

ELL2 5809-mir-128.txt
line 596
ELL2 5811-mir-144.txt
line 415
ELL2 5812-mir-155.txt
line 269

CACNA1A 5809-mir-128.txt
line 608
CACNA1A 5810-mir-143.txt
line 40

CNN3 5809-mir-128.txt
line 611
CNN3 5811-mir-144.txt
line 432

SPOPL 5809-mir-128.txt
line 621
SPOPL 5811-mir-144.txt
line 143

RAPGEF2 5809-mir-128.txt
line 625

RAPGEF2 5811-mir-144.txt
line 378

MARCKS 5809-mir-128.txt
line 626
MARCKS 5810-mir-143.txt
line 200
MARCKS 5811-mir-144.txt
line 493

DUSP5 5809-mir-128.txt
line 633
DUSP5 5811-mir-144.txt
line 617

ARID2 5809-mir-128.txt
line 634
ARID2 5811-mir-144.txt
line 59
ARID2 5812-mir-155.txt
line 15

RUNX1 5809-mir-128.txt
line 658
RUNX1 5811-mir-144.txt
line 139

HEG1 5809-mir-128.txt
line 662
HEG1 5811-mir-144.txt
line 541

GDF6 5809-mir-128.txt
line 667
GDF6 5812-mir-155.txt
line 46

LARP4 5809-mir-128.txt
line 673
LARP4 5810-mir-143.txt
line 118

PDE3B 5809-mir-128.txt
line 690

PDE3B 5811-mir-144.txt
line 443

NAV3 5809-mir-128.txt
line 699
NAV3 5811-mir-144.txt
line 371
NAV3 5812-mir-155.txt
line 203

KIAA0232 5809-mir-128.txt
line 702
KIAA0232 5811-mir-144.txt
line 420

ZC3H12C 5809-mir-128.txt
line 715
ZC3H12C 5811-mir-144.txt
line 180

CDH5 5809-mir-128.txt
line 724
CDH5 5811-mir-144.txt
line 397

GATA2 5809-mir-128.txt
line 732
GATA2 5813-mir-200c.txt
line 25

EDAR 5809-mir-128.txt
line 735
EDAR 5811-mir-144.txt
line 587

MYT1 5809-mir-128.txt
line 746
MYT1 5811-mir-144.txt
line 559

C17orf63 5809-mir-128.txt
line 748
C17orf63 5811-mir-144.txt
line 314

```
TANC2                    5809-mir-128.txt
line   761
TANC2                    5810-mir-143.txt
line   198

PRPF19                   5809-mir-128.txt
line   767
PRPF19                   5810-mir-143.txt
line   164

SURF4                    5809-mir-128.txt
line   772
SURF4                    5813-mir-200c.txt
line    21

KCMF1                    5809-mir-128.txt
line   774
KCMF1                    5810-mir-143.txt
line   182
KCMF1                    5811-mir-144.txt
line   317

ARHGAP26                 5810-mir-143.txt
line     9
ARHGAP26                 5811-mir-144.txt
line    46

PAPD5                    5810-mir-143.txt
line    10
PAPD5                    5811-mir-144.txt
line   251

KRAS                     5810-mir-143.txt
line    21
KRAS                     5812-mir-155.txt
line   102

INADL                    5810-mir-143.txt
line    30
INADL                    5812-mir-155.txt
line   146

USP54                    5810-mir-143.txt
```

line 35
USP54 5811-mir-144.txt
line 386

ALS2 5810-mir-143.txt
line 42
ALS2 5811-mir-144.txt
line 28

FAR1 5810-mir-143.txt
line 51
FAR1 5812-mir-155.txt
line 18

GDF10 5810-mir-143.txt
line 68
GDF10 5811-mir-144.txt
line 138

FAM60A 5810-mir-143.txt
line 124
FAM60A 5811-mir-144.txt
line 195

STRN3 5810-mir-143.txt
line 145
STRN3 5812-mir-155.txt
line 165

CACNA1C 5810-mir-143.txt
line 172
CACNA1C 5812-mir-155.txt
line 267

TRPS1 5810-mir-143.txt
line 186
TRPS1 5812-mir-155.txt
line 134

ABHD2 5810-mir-143.txt
line 194
ABHD2 5812-mir-155.txt
line 245

COL5A2	5810-mir-143.txt
line 236	
COL5A2	5811-mir-144.txt
line 419	
PTGS2	5810-mir-143.txt
line 239	
PTGS2	5811-mir-144.txt
line 451	
RALGPS1	5810-mir-143.txt
line 240	
RALGPS1	5811-mir-144.txt
line 549	
FLJ20160	5810-mir-143.txt
line 263	
FLJ20160	5811-mir-144.txt
line 69	
USP47	5811-mir-144.txt
line 42	
USP47	5813-mir-200c.txt
line 16	
FGF7	5811-mir-144.txt
line 71	
FGF7	5812-mir-155.txt
line 59	
MYBL1	5811-mir-144.txt
line 74	
MYBL1	5812-mir-155.txt
line 78	
SALL1	5811-mir-144.txt
line 86	
SALL1	5812-mir-155.txt
line 244	
ZNF238	5811-mir-144.txt
line 100	
ZNF238	5812-mir-155.txt
line 235	

FAM130A1 5811-mir-144.txt
line 134
FAM130A1 5812-mir-155.txt
line 29

SCN1A 5811-mir-144.txt
line 161
SCN1A 5812-mir-155.txt
line 190

BTBD3 5811-mir-144.txt
line 245
BTBD3 5812-mir-155.txt
line 183

KPNA1 5811-mir-144.txt
line 246
KPNA1 5812-mir-155.txt
line 75

TSHZ3 5811-mir-144.txt
line 256
TSHZ3 5812-mir-155.txt
line 12

MEF2A 5811-mir-144.txt
line 257
MEF2A 5812-mir-155.txt
line 73

SOX1 5811-mir-144.txt
line 288
SOX1 5812-mir-155.txt
line 181

WEE1 5811-mir-144.txt
line 294
WEE1 5812-mir-155.txt
line 88

PKN2 5811-mir-144.txt
line 323
PKN2 5812-mir-155.txt

line 122

RREB1	5811-mir-144.txt
line 422	
RREB1	5812-mir-155.txt
line 156	
ZNRF3	5811-mir-144.txt
line 430	
ZNRF3	5812-mir-155.txt
line 217	
PTP4A1	5811-mir-144.txt
line 483	
PTP4A1	5813-mir-200c.txt
line 26	
RSPO2	5811-mir-144.txt
line 487	
RSPO2	5812-mir-155.txt
line 234	
STXBP5L	5811-mir-144.txt
line 608	
STXBP5L	5812-mir-155.txt
line 56	
NUFIP2	5811-mir-144.txt
line 627	
NUFIP2	5812-mir-155.txt
line 253	
PSKH1	5811-mir-144.txt
line 644	
PSKH1	5812-mir-155.txt
line 231	
